# BnAP2-12 overexpression delays ramie flowering: evidence from AP2/ERF gene expression

**DOI:** 10.3389/fpls.2024.1367837

**Published:** 2024-03-25

**Authors:** Xiaoyang Zhang, Wenxian Peng, Hao Chen, Hucheng Xing

**Affiliations:** ^1^ Agricultural College of Hunan Agricultural University, Changsha, China; ^2^ Ramie Research Institute of Hunan Agricultural University, Changsha, China; ^3^ Changsha Tobacco Company, Ningxiang, China; ^4^ Hunan Key Laboratory of Germplasm Resources Innovation and Resource Utilization Crop Breeding Center, Changsha, China; ^5^ Hunan Provincial Engineering Technology Research Center of Grass Crop Germplasm Innovation and Utilization, Changsha, China

**Keywords:** AP2/ERF, ramie, bioinformatic analysis, gene expression, flowering time

## Abstract

**Introduction:**

The APETALA2/ethylene response factor (AP2/ERF) superfamily plays a significant role in regulating plant gene expression in response to growth and development. To date, there have been no studies into whether the ramie AP2/ERF genes are involved in the regulation of flower development.

**Methods:**

Here, 84 BnAP2/ERF members were identified from the ramie genome database, and various bioinformatics data on the AP2/ERF gene family, structure, replication, promoters and regulatory networks were analysed. BnAP2-12 was transferred into *Arabidopsis* through the flower-dipping method.

**Results:**

Phylogenetic analysis classified the 84 BnAP2/ERF members into four subfamilies: AP2 (18), RAV (3), ERF (42), and DREB (21). The functional domain analysis of genes revealed 10 conserved motifs. Genetic mapping localised the 84 members on 14 chromosomes, among which chromosomes 1, 3, 5, and 8 had more members. Collinearity analysis revealed that 43.37% possibly resulted from replication events during the evolution of the ramie genome. Promoter sequence analysis identified classified cis-acting elements associated with plant growth and development, and responses to stress, hormones, and light. Transcriptomic comparison identified 3,635 differentially expressed genes (DEGs) between male and female flowers (1,803 and 1,832 upregulated and downregulated genes, respectively). Kyoto Encyclopaedia of Genes and Genomes pathway analysis categorised DEGs involved in metabolic pathways and biosynthesis of secondary metabolites. Gene Ontology enrichment analysis further identified enriched genes associated with pollen and female gamete formations. Of the 84 BnAP2/ERFs genes, 22 and 8 upregulated and downregulated genes, respectively, were present in female flowers. Co-expression network analysis identified AP2/ERF members associated with flower development, including BnAP2-12. Subcellular localisation analysis showed that the BnAP2-12 protein is localised in the nucleus and cell membrane. Overexpression BnAP2-12 delayed the flowering time of Arabidopsis thaliana.

**Conclusion:**

These findings provide insights into the mechanism of ramie flower development.

## 1 Introduction

Ramie (*Boehmeria nivea* L. Gaud) is a perennial, persistent, and root fibrous plant species of the Urticaceae family (Huang et al., 2016). It is native to China and is commonly known as “China grass”. According to the growth acreage and fibre production, it is the second most important fibre crop in China, second only to cotton. Additionally, ramie is used as a livestock forage crop with a high level of crude protein (Kandimalla et al., 2016; Fu et al., 2021). Ramie contains flavonoids, chlorogenic acid, and other medicinal compounds, which have medicinal applications for improving heat-clearing haemostasis and detoxification in Traditional Chinese Medicine (Mu et al., 2020); therefore, ramie farming has economic value in China (Zhang et al., 2021). To date, ramie is generally cultivated in South China, along the Yangtze River. To maximise biomass, plants are allowed to flower after three consecutive vegetative harvests.

Plant sex determination is a complex process that is environmentally and genetically determined. Recently, it has been shown that some *sex-determining* are intrinsic factors that influence major sex determination. Some plants have sex chromosomes and their sex-determining genes have been shown to be distributed across these chromosomes. However, most plants have been shown to lack sex chromosomes; in such plants, their sex is determined by multiple genes (Charlesworth, 2016; Harkess and Leebens-Mack, 2017). The relationship between the MADS-box and floral organ development has been most comprehensively studied in *Arabidopsis thaliana*; (Tani et al., 2009) these studies culminated in the “ABCDE model”. In this model, class A genes are mainly AP1, CAL, FUL, and AGL; class B genes are mainly AP3 and PI; class C genes are mainly AG, SPT, and CRC; class D genes are mainly STK (AGL11), SHP1, and SHP2; and class E genes are mainly SEP1 (AGL2, E1), SEP2 (AGL4, E2), and SEP3 (AGL9, E2). (AGL9, E3), and SEP4 (AGL3, E4) (Ma and dePamphilis, 2000). In cucumber, classical genetics has revealed that the primary genes F/f, M/m, and A/a control sex formation (Pawelkowicz et al., 2019). Molecular biology studies have shown the following: that the F (CsACS1G) gene is partially dominant and controls female flower development; the M (CsACS2) gene inhibits stamen development; and the A (CsACS11) gene promotes the development of the carpels. Additionally, these three primary genes are members of the ACC synthase family, which also fully confirms the central role of ethylene in cucumber sex determination(Zhang Y. et al., 2017). There are interactions between related genes involved in sex determination in cucumber, and these interactions may be mediated by ethylene signalling to regulate the expression of related genes (Tao et al., 2018). Moreover, it has been shown that ethylene impacts the expression of CsACS2 and CmWIP1 in cucumber (Chen et al., 2016). Overall, to identify direct mediators (e.g. ERF), it is essential elucidate the potential interactions between ethylene signalling and sex-determining genes.

The APETALA2/ethylene response factor (AP2/ERF) family is an important transcription factor family that plays a vital role in plant growth and development (Klay et al., 2018; Ye et al., 2020; Guyomarc’h et al., 2021; Zhang et al., 2021). To date, members of the AP2/ERF family have been isolated from numerous plants. Structural analysis showed that members of this family are characterised by a DNA-binding domain, transcriptional-regulatory domain, oligomerization site, and nuclear localisation signal domain (Chen J. et al., 2020; Huang et al., 2020). The ERF (ethylene-responsive factor) family is divided into five subfamilies based on the number of AP2 domains and the recognition sequence: AP2 (Apetala2), ERF, DREB (dehydration-responsive element binding proteins), RAV (related to ABI3/VP1), and Soloist (Li et al., 2019). Within the AP2/ERF family, *Arabidopsis thaliana* reportedly has multiple members, including AP2 members, that are involved in flower and seed development (Jofuku et al., 1994; Nakano et al., 2006; Anh Tuan et al., 2016).

Flowering shows the transition of plants from vegetative to reproductive growth (Vijayraghavan, 1996). The mechanisms of flowering have been appropriately elucidated in model plants; however, the mechanisms underlying plant sex determination (male, female, and monoecious features), which are closely related to flowering, remain unclear. The mechanistic elucidation of flower sex determination is crucial for plant breeding (Hassankhah et al., 2020). AP2/ERF genes and other genes have been shown to be associated with flower development. APETALA2 (AP2), an A-class gene, was demonstrated to be involved in flower formation (Jofuku et al., 1994). ERF12 not only regulates flower development in *Arabidopsis*, but it is also associated with meristem identity, floral phyllotaxy, and organ initiation. Additionally, MULTIFLORET SPIKELET1, an orthologous ERF12 from rice, also regulates flower development. Moreover, ERF12 is conserved among angiosperms (Tanaka et al., 2014; Chandler and Werr, 2020). The AP2 gene contributes to plant regeneration and flower development in *Dendrobium officinale* (Zeng et al., 2021). Homologs of AP2 were identified to contribute to reproductive organ development. BABY BOOM regulates somatic embryogenesis in rapeseed. ANT is involved in the development of ovule development in *A. thaliana*. IDS1 and SID1 regulate the initiation of corn flower meristems to determine the fate of meristem cells (Chuck et al., 2008; Zeng et al., 2021). All these data indicate that AP2/ERF members have pleotropic effects on plant development.

Ramie is a monoecious plant. Given that ramie is traditionally reproduced via vegetative propagation for fibre, little is known about the molecular and genetic mechanisms that underlie is its sexual development. Presently, the sexual reproduction of ramie is more advantageous than asexual reproduction, as it yields offspring that exhibit more variation. To further optimise the sexual reproduction of this plant, and to select flowerless varieties, it is necessary to identify the genes related to flowering and sex differentiation. The genomes of Boehmeria nivea (variety: Zhongsizhu No. 1) have been sequenced and assembled; these sequences provide an appropriate platform to identify genes involved in flower differentiation and development (Wang et al., 2021). In this study, we systematically conducted genome-wide identification of the AP2/ERF gene family in *B. nivea* by analysing the sequence conservation, gene location, gene structure, and evolutionary relationships and introducing BnAP2-12 into *A, thaliana*.

## 2 Materials and methods

### 2.1 Plant materials

Two ramie cultivars, GBN08 and GBN09, were used as plant material; GBN09 is a female germplasm, whereas GBN08 is a monoecious germplasm developing male flowers in the lower part of the plant and female flowers in the upper part (**Supplementary Figure S1**). In 2015, seedlings of two cultivars were planted in the Yunyuan of Hunan Agricultural University. The rows of plants were spaced 50 cm apart. Field management included weeding and cutting. The growth and flower development of plants were observed and recorded every week. On September 20, 2019, flowers of the same phase were collected from six individual blooming ramie plants (including young leaf, skin, and stem) and pooled as one biological sample. Six biological samples for each phase were collected. All samples were immediately frozen in liquid nitrogen and stored at −80°C for RNA extraction.

### 2.2 Bioinformatics analysis for ramie AP2/ERF gene family identification

The ramie genome was downloaded from the NCBI Centre for Biotechnology Information (https://www.ncbi.nlm.nih.gov/bioproject/PRJNA663425) database. Protein, CDS Sequence, and gff3 files were obtained from Wang et al. (2021). The Hidden Markov Model profile (PF00847) of the AP2/ERF domain in the Pfam database was obtained for searching potential genes in ramie using the HMMER 3.0 software with default parameters (Potter et al., 2018). The obtained ramie AP2/ERF candidate genes were further confirmed using Pfam (http://pfam.xfam.org/) and the NCBI Conserved Domain Database (CDD, https://www.ncbi.nlm.nih.gov/). All predicted gene lengths, coding sequences, and protein lengths were determined using NCBI (https://www.ncbi.nlm.nih.gov). Expasy ProtParam (https://web.expasy.org/protparam/) was used to predict the physical and chemical parameters of BnAP2/ERF proteins; this information included the number of amino acids, molecular weight, theoretical pI, instability index, aliphatic index, and grand average of hydropathicity (GRAVY). The ORFs of all BnAP2/ERF genes were analysed using the find ORF programme (https://github.com/vsbuffalo/findorf). The subcellular localisation of BnAP2/ERF proteins were predicted using Cell-PLoc2.0 (http://www.csbio.sjtu.edu.cn) [Chou and Shen, 2008]. The BnAP2/ERFs proteins were aligned using Clustal (version: X2.0, University College Dublin, Dublin, Ireland) [Larkin et al., 2007]. A phylogenetic tree of BnAP2/ERFs was constructed using comparison and alignment functions in the muscle software (Pei, 2008) in ramie and *Arabidopsis*. The maximum likelihood tree (ML tree) developed using iqtree with 1,000 bootstraps and default parameters (http://iqtree.cibiv.univie.ac.at/) [Trifinopoulos et al., 2016]. The amino acid sequences of BnAP2/ERF were submitted to the MEME website (http://meme-suite.org/) [Bailey et al., 2015] for conserved amino acid sequence analysis. The results were plotted using TBtools (Chen C. et al., 2020). The locations of introns and exons of ramie AP2/ERF genes were analysed using GSDs (http://gsds.gao-lab.org/) [Hu et al., 2015]. The localisation of these genes in the chromosomes was determined using MapChart software (Voorrips, 2002). A total of 2,000 bp sequences upstream of the start codon (ATG) in genomic DNA sequences were deposited in Plant CARE (http://bioinformatics.psb.ugent.be/webtools/plantcare/html/) to identify the cis-acting elements of AP2/ERF genes (Lescot et al., 2002). The results were visualised using TBtools (Chen C. et al., 2020).

### 2.3 Collinearity analysis and Ka/Ks calculation

Collinearity was analysed using MCScanX between ramie and *Arabidopsis*, rice, ramie, and cotton (Wang et al., 2012). The Simple Ka/Ks Calculator module in TBtools was used to determine the ratios of synonymous (Ks) and non-synonymous (Ka) nucleotide substitutions (Ka/Ks) in homologous gene pairs (Chen J. et al., 2020). The divergence time was calculated using the formula T = Ks/2r, where Ks is the ratio of synonymous and r is equal to 3.39 × 10^−9^ synonymous substitutions per site per year (Wu et al., 2019).

### 2.4 RNA sample preparation and sequencing

Total RNA was extracted using TRIzol Reagent (Life Technologies, USA) and the RNA quality was evaluated using an Agilent 2100 BioAnalyzer (Agilent, USA). RNA-Seq libraries were constructed using the RNA Library Prep Kit for Illumina (NEB, USA) according to the manufacturer’s instructions. Library quality was evaluated using the Agilent Bioanalyzer 2100 system. The libraries were sequenced with the Illumina HiSeq 2000 platform (Illumina, USA) to generate 100 bp paired end reads (Berry Genomics, Beijing). After removing low quality sequences, the resulting high-quality sequences were assembled into unigenes using the short reads assembling programme Trinity with min kmer cov set to two and all other parameters set to default. All unigenes were matched to the ramie genome (PRJNA663425). The Nr, Swiss Prot, KO, and Gene Ontology (GO) databases were used to annotate gene functions.

### 2.5 Differential expression and functional enrichment analysis

High quality RNA-seq reads of both male and female flowers were aligned to the “ZSZ No.1” (*Boehmeria nivea*) genome. Differentially expressed genes (DEGs) GBN08 and GBN09 for male and female flowers, respectively, were identified for expression profiling analysis. The FPKM values of DEGs were normalised using a variance stabilising transformation (VST). Differential expression was analysed using the DESeq2 software between the two groups with default FDR < 0.05 and absolute log-fold-change > 1 (Love et al., 2014). DEGs were submitted to the GO and Kyoto Encyclopaedia of Genes and Genomes (KEGG) websites for enrichment analysis. A two-tailed Fisher’s exact test was employed to identify enrichment. The total number of genes used for GO analysis was 8192, and the total number of genes used for KEGG analysis was 3732. A corrected *p*-value < 0.05 was used to indicate significant expression of genes between the sexual flowers.

### 2.6 Expression analysis of BnAP2/ERF

To better determine the expression patterns of AP2/ERF, the transcriptional quantity and gene expression count matrices were obtained using feature counts (Li et al., 2023). The FPKM values of BnAP2/ERF were used to represent the expression level. All FPKM values were normalised with log2 (FPKM+1), and the resulting data were used to construct heatmaps using TBtools (Chen C. et al., 2020). After the transcriptional levels were compared between samples, candidate BnAP2/ERF genes were selected for qRT–PCR analysis using the Maxima SYBR Green qPCR Master Mix (Invitrogen Corp, Carlsbad, CA, USA). All total RNA of samples (female flower, male flower, young leaf, young skin, and young stem) were exacted using a plant RNA extraction kit (Vazyme, Nanjing, China) according to the manufacturer’s instructions. The high-quality RNA samples were used to synthesise the first strand cDNA using a reverse transcription kit (Vazyme, Nanjing, China). Primers were designed using Premier 5.0 (**Supplementary Table S1**) and synthesised via Sangon Biotech (Shanghai, China). The resultant cDNA was used as a template for qPCR analysis with the Light Cycler 96 Real Time PCR (Roche, Swiss) using the qPCR Super Mix (TransGen Biotech, Beijing, China). The housekeeping BnActin gene was used as the reference. Three biological replicates were completed, and each experiment was repeated three times. The qRT–PCR data were processed using the 2^-(△△CT)^ method. DPS (version 9.0) was used for one-way analysis of variance (ANOVA).

### 2.7 Co-expression network analysis

All DEGs of AP2/ERF were used for Pearson correlation analysis. Additionally, DEGs with FDR absolute correlation > 0.8 and p-Value <0.0001 were used for co-expression analysis to develop a co-expression network based on gene expression profiles. The genes directly interacting with AP2/ERF were visualised using Cytoscape v3.9.1. RNA-Seq reads were obtained from different tissues of ramie GBN-08 and GBN-09 cultivars. The reads were then aligned to the ramie genome and normalised using VST. Genes with VST > 3 were differentially regulated. Next, all samples were clustered using Euclidean distance, and all genes were scaled and clustered using Pearson correlation. Finally, mutual information (MI) and contextual linked likelihood (CLR) algorithms were used for co-expression network analysis. Regardless of the cell type used, the co-expression network only depended on the gene expression patterns. Hubs in the transcriptomes of different sexual flowers were selected based on the degree rank obtained for each gene in cytoHubba (Chin et al., 2014). The top 50 nodes ranked by degree were defined as “hubs”. All proposed gene co-expression networks were generated using a co-expression threshold of 12. The networks were visualised using Cytoscape v3.9.1.

### 2.8 Cloning of BnAP2-12 cDNA and phylogenetic analyses

The first strand cDNAs were used as the template for PCR. A pair of primers (forward AP2-12-SF: CGGGATCCATGGCAAAGATCTCACGG and reverse AP2-12-SR: CCCCCGGGTTAATCTAGGACTCCTTCACAATG) were designed to amplify open reading frame (ORF) using the Primer Premier 5.0 software. The PCR amplification programme comprised the following: 5 min at 95 °C, 33 cycles (30 s at 95 °C, 30 s at 57 °C, and 90 s at 72 °C), and 72 °C extension for 10 min. The PCR product was examined on a 1.5% agarose gel via electrophoresis. The band was cut from the gel for purification using an Easy Pure Quick Gel Extraction Kit. The purified cDNA was cloned to a pEASY-Blunt Zero vector (Trans Gen) to create a fusion construct, namely pEASY-BluntZero-BnAP2-12. The recombinant vector was transformed into a Trans10 Phage-Resistant Chemically Competent Cell (manufactured by Trans Gen Biotech Company). A positive colony was obtained to purify plasmids for sequencing at Shanghai Sheng gong Cooperation (Shanghai, China). After the full-length nucleotide sequence was confirmed, it was submitted to NCBI for alignment analysis. BLASTp was used for protein sequence alignment and domain prediction. MEGA (version 5.05) was used for sequence analysis. The alignment of multiple sequences was performed using ClustalW. Subsequently, a phylogenetic tree was constructed using the neighbour-joining method, with 1000 bootstrap. BnAP2-12 and 10 other AP2 factors from different plants (identified from the NCBI GenBank database) were included in this phylogenetic analysis. A tree was constructed using Kimura’s two-parameter model.

### 2.9 Subcellular localisation of BnAP2-12 protein

A transient expression vector was constructed to express BnAP2-12 and localise it in cells. Briefly, a pCAMBIA1302-GFP vector was digested using NcoI and SpeI. The linearly digested product was purified for ligation. The ORF of BnAP2-12 lacking its stop codon and the digested vector was ligated with T4 DNA ligase. The resulting vector included 35S∷BnAP2-12-GFP. The recombinant binary vector was introduced into Agrobacterium GV3101. A positive colony selected from antibiotic-resistant medium was used to infect *Nicotiana benthamiana*, as previously reported (Chai et al., 2014). Additionally, pCAMBIA1302-GFP was used a control. After the infected leaves were incubated for 48 h, they were examined with a laser confocal microscope (LSM780, Zeiss).

### 2.10 Genetic transformation of *A. thaliana*

Seeds of *A. thaliana* Col were stratified at 4 °C and then germinated in pot soil in a controlled environment chamber. Seedlings were grown in the same conditions for flowering. The ORF of BnAP2-12 was cloned into the binary vector pBI121 to generate new recombinant binary vector pBI121BnAP2-12, in which BnAP2-12 was driven by a 35S promoter. The new binary vector was introduced into *Agrobacterium tumefaciens* GV3101. For activation of CV3101, a positive colony was inoculated to 200 mL of YEP liquid medium in a 500 ml E-flask, which was placed on a shaker at 250 rpm overnight. When the OD_600_ of GV3101 was approximately 1.5, the culture was harvested for 10-min centrifugation at 3000 × g and 4°C. After the supernatant was removed to a waste container, the pellet was suspended in 1/2MS liquid medium supplemented with 0.03% silwet-77 surfactant in a 500 mL beaker. The suspension was adjusted to a final OD600 of 0.8. The half-blooming inflorescences were immersed in the Agrobacterium suspension for 1 min in the dark, followed by a 24-h incubation. This infiltration process was repeated twice. Infiltrated plants were grown in the same growth chamber to harvest seeds. Seeds were selected on MS medium supplemented with 50 mg/L kanamycin to screen transgenic plants. Positive resistant plants were grown in the same growth conditions to screen T2 plants. Seeds of T2 plants were germinated to phenotype and the development of transgenic plants in the same growth condition was photographed.

## 3 Results

### 3.1 Flowering characteristics of GBN08 and GBN09

**Figure 1:**
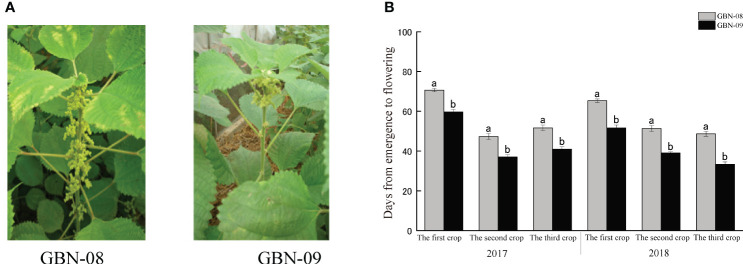
Inflorescence of male and female ramie in field. **(A)** blooming female GBN-08 and male GBN-09 inflorescences; **(B)** Flowering interval of GBN-08 and GBN-09.

Wild type ramie is a short photoperiod plant. In the Yangtze River Basin, ramie plants generally start to flower in early autumn and blossom in middle to late September. GBN08 and GBN09 are two ramie cultivars for fibre production. Unlike the wild type, their flowering time is not influenced by the photoperiod. The two cultivars are generally cropped three times per year in the Yangtze River Basin. Both bloom from April to June (the first cropping season), July to August (the second cropping season), and September to October (the third cropping season). The main difference in flowering time between the cultivars is that GBN08 generally starts blooming 16–17 d after GBN09 (**Figure 1**).

Typically, the initiation of inflorescence emergence begins in the middle region of ramie plants. Then, plants start the differentiation of male and female flowers. The lower part develops as male flowers, while the upper part develops as female flowers. Simultaneously, some inflorescences develop monoecious flowers. The two cultivars follow these features. Our examination of inflorescences revealed that the numbers of inflorescences were similar between the two cultivars. The main difference was that GBN-09 started to develop inflorescences in lower nodes than GBN-08 (**Supplementary Table S1**).

### 3.2 Identification and sequence characterizations of BnAP2/ERF genes in ramie

We used the HMMER software to identify 84 AP2/ERF from the published genome of ramie. Based on the sequence and conserved domain features, the 84 members were composed of four subfamilies: AP2 (18 members), ERF (41 members), RAV (3 members), and DREB (21 members) (**Supplementary Figure S1**). The ORF length of the 84 BnAP2/ERF ranged from 615 to 2322 bp. The molecular weights of the 84 BnAP2/ERF proteins ranged from 22.06 to 83.35 kDa, and the theoretical pIs ranged from 4.59 to 10.80. The prediction of subcellular localisation suggested that most BnAP2/ERF proteins are localised in the nucleus, with a few localised in the cytoplasm and one (BnAP2-7) localised in the chloroplast (**Table 1**).

**Table 1:** Characteristics of putative AP2/ERF genes in ramie.

Subfamily	Group	Gene rename	ORF(bp)	Proteinlength (aa)	MW(kD)	pI	Subcellular Localization
ERF	B3	BnERF-1	882	293	32.03	6	Nucleus
		BnERF-2	1020	339	35.64	8.39	Nucleus
		BnERF-3	756	251	27.13	6.39	Nucleus
		BnERF-4	1422	474	51.81	5.64	Nucleus
		BnERF-5	798	265	29.92	5.82	Nucleus
		BnERF-6	888	295	32.82	5	Nucleus
		BnERF-7	742	248	26.91	5.52	Nucleus
		BnERF-8	615	204	22.26	5.15	Nucleus
		BnERF-9	756	251	27.95	5.8	Nucleus
		BnERF-10	969	322	35.79	7.78	Nucleus
		BnERF-11	885	294	32.5	9.19	Nucleus
		BnERF-12	801	266	29.23	5.9	Nucleus
		BnERF-13	984	327	34.45	5.9	Nucleus
	B2	BnERF-14	1179	392	43.78	4.86	Nucleus
		BnERF-15	870	289	32.25	8.5	Nucleus
		BnERF-16	708	235	26.59	5.67	Nucleus
	B4	BnERF-17	1014	337	36.54	6.99	Nucleus
		BnERF-18	762	253	28.75	8.54	Nucleus
		BnERF-19	1137	378	41.78	6	Nucleus
		BnERF-20	1314	437	47.49	6.94	Nucleus
	B6	BnERF-21	792	263	28.49	7.7	Cytoplasm. Nucleus.
		BnERF-22	675	224	24.14	9.19	Nucleus
		BnERF-23	741	240	27.21	7.21	Nucleus
		BnERF-24	630	260	29.09	9.71	Nucleus
		BnERF-25	630	209	22.95	5.74	Nucleus
		BnERF-26	711	236	26.68	6.14	Nucleus
		BnERF-27	888	295	32.1	8.98	Nucleus
		BnERF-28	852	283	29.28	5.78	Cytoplasm. Nucleus.
		BnERF-29	882	293	32.34	5.76	Cytoplasm. Nucleus.
		BnERF-30	1263	420	46.09	4.59	Cytoplasm. Nucleus.
	B5	BnERF-31	936	311	33.98	5.16	Nucleus
		BnERF-32	1023	340	37.63	5.33	Cytoplasm. Nucleus.
		BnERF-33	720	239	26.28	7.77	Nucleus
	B1	BnERF-34	1155	384	42.48	5.29	Nucleus
		BnERF-35	1239	412	45.4	6.97	Nucleus
		BnERF-36	978	325	34.59	10.8	Nucleus
		BnERF-37	702	233	24.16	8.97	Nucleus
		BnERF-38	804	267	28.56	9.43	Nucleus
		BnERF-39	663	220	23.77	9.18	Nucleus
		BnERF-40	663	220	23.45	7.67	Nucleus
		BnERF-41	774	257	28.43	9.82	Nucleus
		BnERF-42	495	251	26.52	4.91	Nucleus
DREB	A4	BnDREB-1	714	237	25.64	5.97	Cytoplasm. Nucleus.
		BnDREB-2	2085	695	75.84	5.93	Cytoplasm. Nucleus.
		BnDREB-3	642	213	22.06	5.64	Cytoplasm. Nucleus.
		BnDREB-4	666	221	23.45	5.97	Nucleus
		BnDREB-5	795	264	27.87	4.98	Nucleus
		BnDREB-6	933	310	32.86	5.69	Cytoplasm. Nucleus.
		BnDREB-7	843	280	30.19	5.11	Nucleus
		BnDREB-8	906	301	33.08	5.61	Nucleus
		BnDREB-9	879	292	31.89	5.54	Cytoplasm. Nucleus.
	A5	BnDREB-10	825	274	31.01	4.69	Cytoplasm. Nucleus.
		BnDREB-11	639	212	23.04	5.13	Cytoplasm. Nucleus.
		BnDREB-12	636	211	23.01	4.67	Cytoplasm. Nucleus.
		BnDREB-13	840	279	30.89	5.97	Cytoplasm.
	A6	BnDREB-14	999	332	36.52	7.82	Nucleus
		BnDREB-15	969	322	35.27	9.14	Nucleus
		BnDREB-16	1215	404	44.69	6.52	Nucleus
		BnDREB-17	1323	440	49.39	6.3	Nucleus
	A2	BnDREB-18	1128	375	41.94	6.36	Cytoplasm.
		BnDREB-19	1068	452	49.58	5.97	Cytoplasm. Nucleus.
	A3	BnDREB-20	1005	334	35.44	8.12	Nucleus
		BnDREB-21	996	331	37.02	5.56	Nucleus
AP2		BnAP2-1	2268	755	83.35	7.23	Nucleus
		BnAP2-2	2322	773	82.9	6.46	Cytoplasm. Nucleus.
		BnAP2-3	2217	738	79.69	6.84	Nucleus
		BnAP2-4	1908	635	69.98	6.36	Cytoplasm. Nucleus.
		BnAP2-5	1668	555	59.6	6.84	Nucleus
		BnAP2-6	1704	567	60.95	6.75	Nucleus
		BnAP2-7	1494	498	54.17	8.92	Chloroplast. Cytoplasm.
		BnAP2-8	1284	427	47.64	8.91	Cytoplasm. Nucleus.
		BnAP2-9	1320	439	50.34	6.06	Cytoplasm. Nucleus.
		BnAP2-10	1218	405	44.11	5.5	Cytoplasm. Nucleus.
		BnAP2-11	1149	386	43.25	7.79	Cytoplasm. Nucleus.
		BnAP2-12	1113	370	41.15	6.8	Cytoplasm. Nucleus.
		BnAP2-13	1482	501	54.84	6.66	Nucleus
		BnAP2-14	1287	428	47.05	9.04	Cytoplasm. Nucleus.
		BnAP2-15	1602	533	56.86	6.37	Cytoplasm. Nucleus.
		BnAP2-16	897	298	33.74	8.5	Cytoplasm.
		BnAP2-17	807	268	29.14	8.35	Nucleus
		BnERF-18	705	234	26.04	6.72	Cytoplasm. Nucleus.
RAV		BnRAV-1	1236	411	43.45	8.85	Nucleus
		BnRAV-2	1110	369	41.63	8.12	Nucleus
		BnRAV-3	1176	391	43.53	5.58	Nucleus

To localise the conserved motifs of AP2/ERFs, the MEME programme and TB tools software were employed to analyse the full-length protein sequences. This analysis identified 10 motifs from the 84 members (**Supplementary Figure S2**). Motifs 1, 2, and 3 existed in all of them. The conserved amino acid sequences of motifs 1 and 2 were RAYD and YRG in the AP2 domain, respectively. Additionally, based on the genome sequences, exon and intron gene sequences were analysed. The resulting data showed that 41 genes (49.4%) did not have introns; the number of exons ranged from 1–10.

The distributions of 84 AP2/ERF genes on chromosomes were analysed, and the resulting data revealed that each chromosome has at least two genes. Chromosome 1 has nine genes, and chromosomes 3, 5, and 8 each have eight genes. Gene cluster analysis revealed two gene clusters on chromosomes 3 and 5 and one cluster on chromosomes 7, 8, 12, and 13 (**Supplementary Figure S3**).

Cis-elements of promoters of the 84 members were analysed. The results revealed 22 primary types, consisting of 2,134 cis-elements, associated with plant growth and development, abiotic and biotic stress responses (anaerobic induction, anoxic specific inducibility, defence and stress responsiveness, drought-inducibility, low-temperature responsiveness, and salt stress), wound-response, and plant hormone responses (including ABA responsiveness, auxin-responsiveness, GA responsiveness, MeJA-responsiveness, and salicylic acid responsiveness) (**Supplementary Figure S4**, **Supplementary Table S2**). Among these, the light-responsive elements existed in all genes. The hormone-, stress-, and growth and development-responsive elements existed in 83, 82, and 65 genes, respectively. The G-box (294) and Box 4 (237) of light-responsive elements were present in the promoters of 74 and 79 members, respectively. Abscisic acid response elements (253) existed in the promoters of 73 BnAP2/ERF genes. These results support the cis-element types found in other plant species (Wong et al., 2017; Jiang et al., 2022; Qiu et al., 2022; Wan et al., 2022).

### 3.3 Collinearity analysis and Ka/Ks calculation

**Figure 2:**
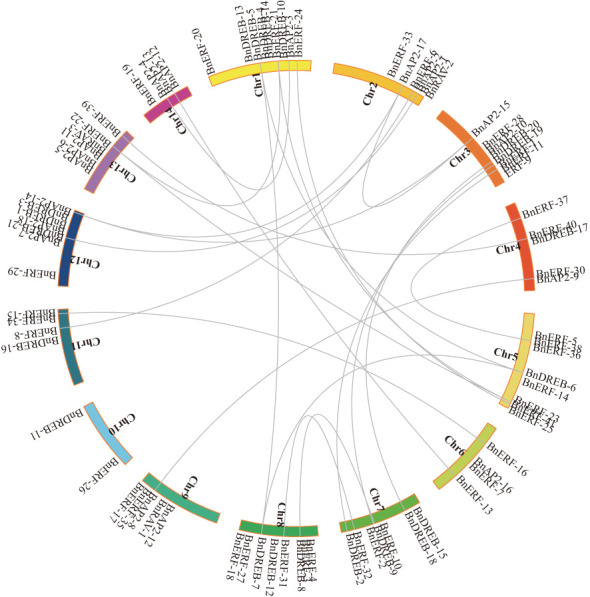
Collinearity of AP2/ERF genes in Ramie. AP2/ERF genes were mapped into different chromosomes in Ramie. The chromosome number is indicated on the inside. Lines connecting two chromosomal regions indicate synteny blocks in the Ramie genome.

The co-linearity of the AP2/ERF gene family in the ramie genome was analysed to understand potential relationships and replication events. The results showed that except for the single-copy of BnAP2-16 in the ramie genome, 34 genes could be derived from replicative, non-replicative, or conservative translocations; 5 genes could be derived from small-scale translocations or from tandem replication and the insertion of some other genes; and 37 genes could be derived from whole-genome replication or fragment replication (**Figure 2**).

Several AP2/ERF genes in the ramie exhibited strong syntenic relationships with genes from various species (**Supplementary Figure S5**). Ramie has more chromosomes than *Arabidopsis*, which results in relatively scattered gene distributions. Furthermore, fragments were present on chromosome 10 that did not have a collinear relationship with *Arabidopsis* (**Supplementary Figure S5A**). The number of collinear genes between ramie and rice was considerably lower than that between *Arabidopsis* and rice (**Supplementary Figure S5B**). Ramie, being a dicotyledonous plant, exhibited significantly higher orthologous gene pairs with other dicotyledons (e.g. *Arabidopsis thaliana*) compared to monocotyledons (e.g. *Oryza sativa*). Some collinear genes were exclusively present between ramie and other dicotyledons. For instance, the number of collinear genes and their distribution on chromosomes between ramie and jujube (**Supplementary Figure S5C**) or cotton (**Supplementary Figure S5D**) exceeded those between ramie and rice or *A. thaliana*. Additionally, ramie is a fibre crop, the orthologous gene pairs of ramie and other fibre crop (cotton) were significantly higher than those of a non-fibre crop (e.g., jujube).

Ka/Ks represents the ratio between non-synonymous (Ka) and synonymous (Ks) substitutions, which can determine whether the gene encoding the protein is under selection pressure. Ka/Ks > 1 denotes positive selection, Ka/Ks ≈ 1 denotes neutral selection, and Ka/Ks < 1 denotes purifying selection (Qiu et al., 2022). All Ka/Ks values of BnAP2/ERF were < 1. The BnERF-03/01 and BnERF-05/01, Bni06T009529.1 and Bni14T018819.1, Bni03T004459.1 and Bni07T010165.1, Bni03T004495.1 and Bni07T010642.1, and Bni02T003428.1 and Bni12T016797.1 duplicate pairs comprised higher degrees of sequence divergence, indicating a greater evolutionary distance. The divergence time of each pair was approximately 113–348 MYA in ramie (**Supplementary Table S3**).

### 3.4 Correlation of ramie transcript profiles for different gender flowers

**Figure 3:**
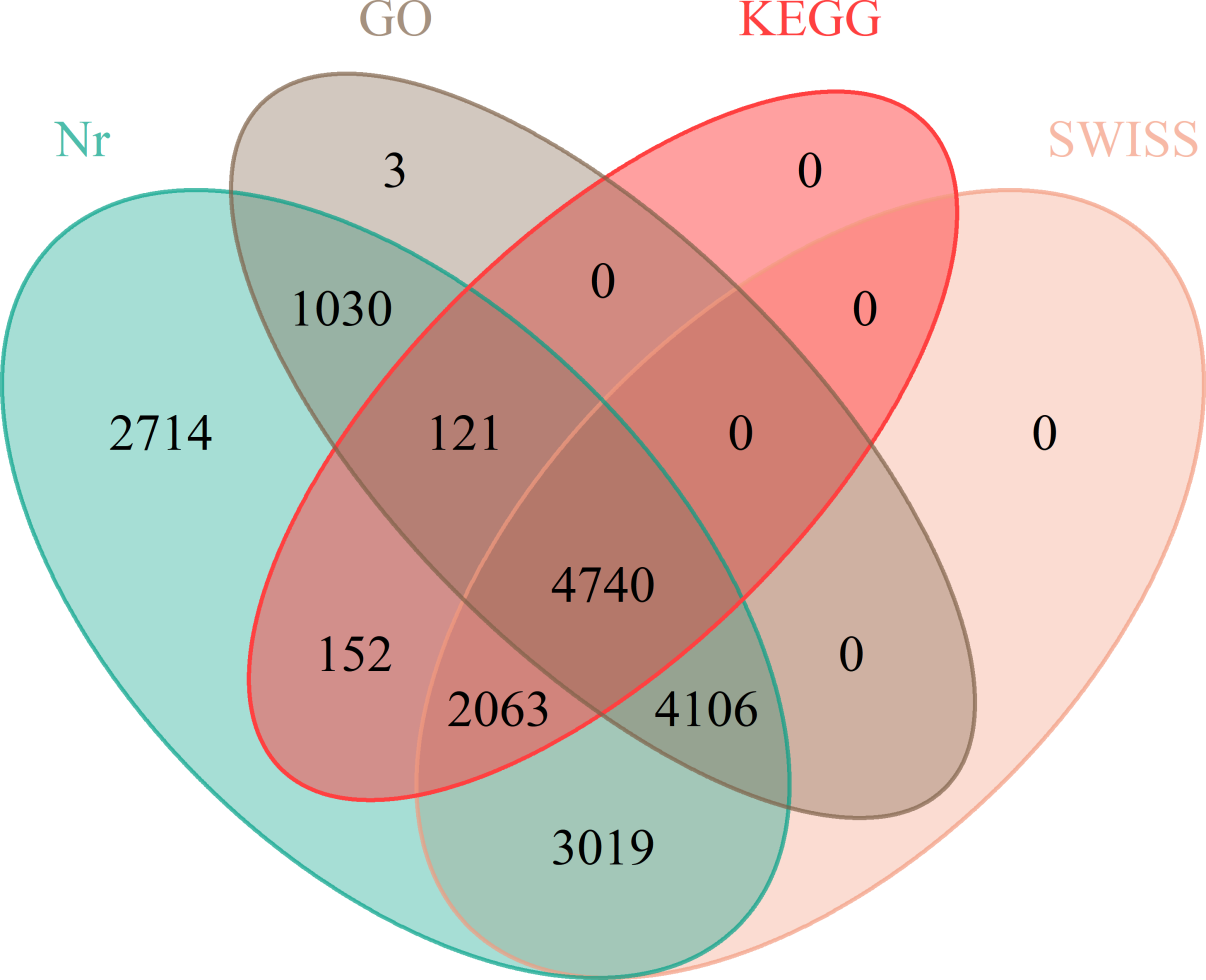
Venn diagram annotations of transcripts.

Given that the genomic information regarding the floral differentiation of ramie is limited, we completed RNA-seq to assemble the transcriptomes of flowers of different sexes. The total numbers of unique mapped Reads in the RNA-seq libraries ranged from 36,763,885 to 46,946,564, in which more than 71% of the paired reads were mapped to the genome. The percentages of Q20 and Q30 bases in the raw data averaged 95.39% and 90.289%, respectively (**Supplementary Table S4**). GBN09 germplasm had only female flowers, whereas GBN08 germplasm had both male and female flowers. The assembled transcriptomes revealed DEGs associated with sexual differentiation between GBN08 and GBN09. Further analysis of DEGs at Nr, KEGG, Swiss-Prot, and GO revealed 17,945 (Nr), 7,076 (KEGG), 13,928 (Swiss-Prot), and 10,000 (GO) genes associated with different functions (**Figure 3**).

**Figure 4:**
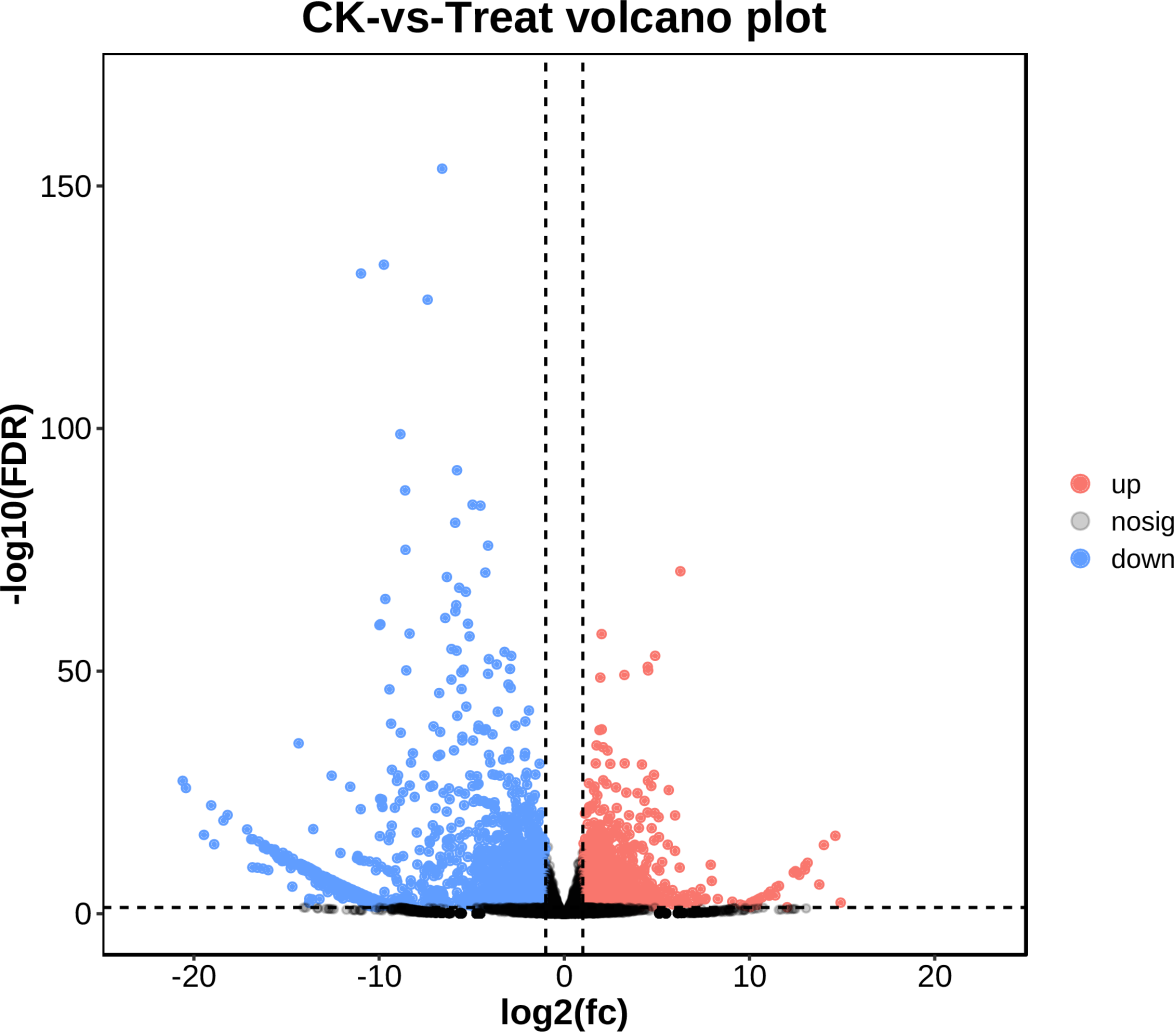
Volcano map of differentially expressed genes.

**Figure 5:**
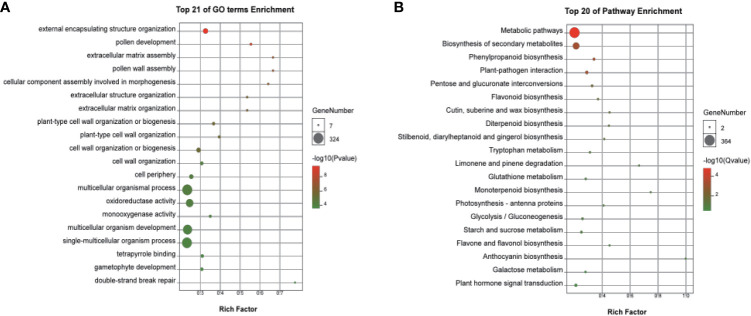
Significantly enriched GO and KEGG pathway annotations of DEGs between male and female flowers. **(A)** Top 21 of GO terms enrichment and **(B)** Top 20 of KEGG pathway enrichment. GO terms analysis of DEGs in **(A)** male vs. female or KEGG pathway analysis of DEGs in **(B)** male vs. female. The size of each circle represents the number of genes, and colour represents the p-value **(A)** or Q-value **(B)**. P-value and Q-value ≤ 0.05 indicate significant enrichment.

Based on |log2 Foldchange| > 1, p.adj < 0.05 parameters, 3,635 DEGs were identified, including 1,803 upregulated and 1,832 downregulated DEGs in the two cultivars (**Figure 4**, **Supplementary Table S5**). A classification with BLAST hit using Blast2GO clustered the DEGs into biological processes, molecular functions, and cellular components. For biological processes, multiple DEGs were revealed to associate with metabolic, cellular, and single-organism processes. For cellular components, most DEGs were clustered in cell, cell part, organelle, membrane, and membrane part. DEGs of molecular functions were enriched in catalytic activity, binding, and others. Enrichment analyses revealed multiple DEGs related to flower development, including reproductive processes (**Supplementary Figure S6**). In particular, the top 20 functions from GO included pollen development (GO:0009555), pollen wall assembly (GO:0010208), cellular component assembly involved in morphogenesis (GO:0010927), and gametophyte development (GO:0048229), which were significantly enriched in the female flowers of GBN09 compared with that in the male flowers of GBN08 (**Figure 5A**).

KEGG functional enrichment analysis for DEGs revealed genes associated with the differentiation of male and female flowers. The main metabolic differences in the two sexual flowers were characterised by carbohydrate metabolism, lipid metabolism, biosynthesis of secondary metabolism, signal transduction, and environmental adaption (**Figure 5B**; **Supplementary Figure S7**). Several enriched DEGs were related to plant hormone signal transduction during the development of the sexual flowers, such as auxin responsive genes, ethylene signal transduction pathway genes, ethylene-responsive genes, and gibberellin responsive genes among the DEGs related to plant hormone signal transduction during the development of the male or female flowers (**Figure 5B**; **Supplementary Table S5**).

The DEGs had 171 transcription factors (TFs), including 30 AP2/ERFs, 28 MYBs, 17 bHLHs, 14 HD-ZIPs, 12 WRKYs, and 72 other family members. Of these, female flowers of GBN09 expressed 22 ethylene-responsive TFs with a much higher level and 8 ethylene-responsive TFs with a much lower level than the male flowers of GBN08. Additionally, multiple DEGs were related to ethylene biosynthesis and signal transduction, including EFE (Ethylene-forming enzyme; Bni05T007712), ERS (ethylene response sensor; Bni06T008526), ETO (Ethylene-overproduction protein; Bni02T002774), EIN3 (ETHYLENE INSENSITIVE 3; Bni03T004274), and Weak ethylene-insensitive protein (Bni05T006945). These genes were enriched during flower development, showing that ethylene may play an essential a role in regulating female and male flower development in ramie (**Supplementary Table S5**).

### 3.5 Expression pattern of BnAP2/ERF genes among ramie tissues

**Figure 6:**
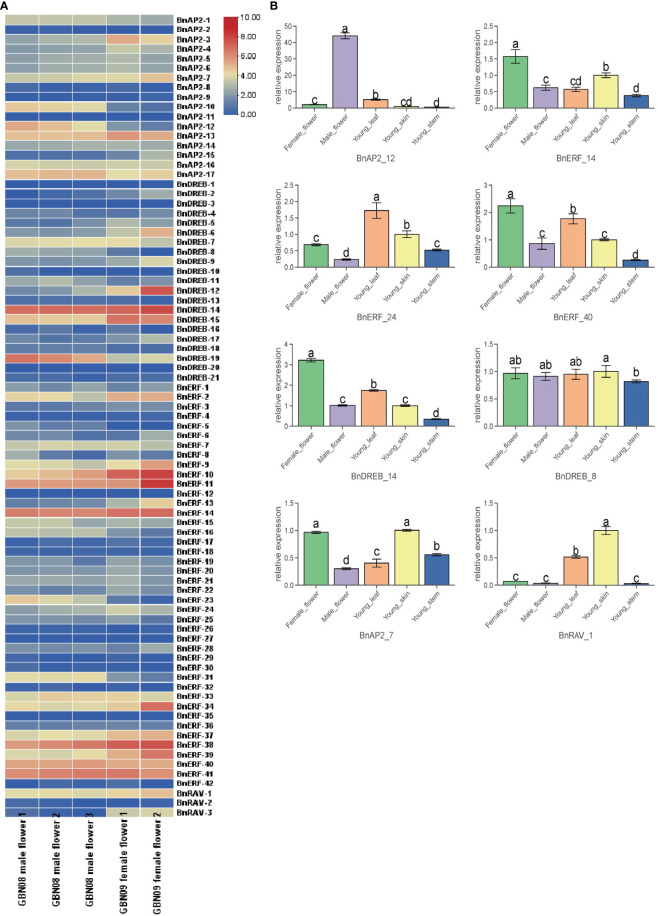
Expression profiles of BnAP2/ERF. **(A)** the expression profile of 84 *BnAP2/ERF* in different flower tissues. The colours from blue to red correspond to gene expression from a low to a high level, respectively. **(B)** qRT-PCR data show the relative expressions of eight *BnAP2/ERF* genes in different tissues. Bars labelled with different low case letters indicate significant differences at the P < 0.05 level (n=3).

Based on the abovementioned transcriptomes, BnAP2/ERF genes were selected to understand their potential roles in ramie flower development. The expression profiles of BnAP2/ERF genes were analysed in different tissues. The results showed that four genes (BnERF-38, BnERF-11, BnERF-10, BnDREB-14) were highly expressed in all tested tissues (average FPKM > 100; **Figure 6A**; **Supplementary Table S6**), and the expression of five genes (BnAP2-11, BnDREB-3, BnDREB-20, BnERF-12, and BnERF-27) was not detected in any tissues (FPKM = 0). The expression of other members was differentially detected in different tissues. BnAP2-10, BnAP2-12, BnERF-16, BnERF-23, BnERF-31, and DREB19 had a higher expression level in the male flowers of GBN08 than in the female flowers of GBN09. In contrast, BnERF-2, BnERF-10, BnERF-11, BnERF-13, and BnDREB-12 had higher expression in the female flowers of GBN09 than in the male flowers of IBN08. This suggests that these genes may be associated with the differentiation or development of male or female flowers. Overall, these findings indicate that the expression of BnAP2/ERF genes is related to the sexual differentiation of ramie flowers.

The expression profiles of 8 BaAP2/ERFs, randomly selected from the 84 members, were examined with qRT–PCR to validate the results from RNA-seq (**Figure 6B**). The qRT–PCR data were similar to those of expression profiles revealed by RNA-seq. For example, the expression level of BnAP2-12 was 40 times higher in male flowers than it was in female flowers. The expression levels of BnERF-40 and BnDERB-14 were significantly higher in female flowers than in other tissues. The expression level of BnAP2-7 was similar in all tissues. The expression of BnERF-24 was higher in young leaves than in stem skins. The expression of BnDREB-8 was significantly lower in the stem than in skin.

### 3.6 Network analysis of DEGs in male and female flowers

**Figure 7:**
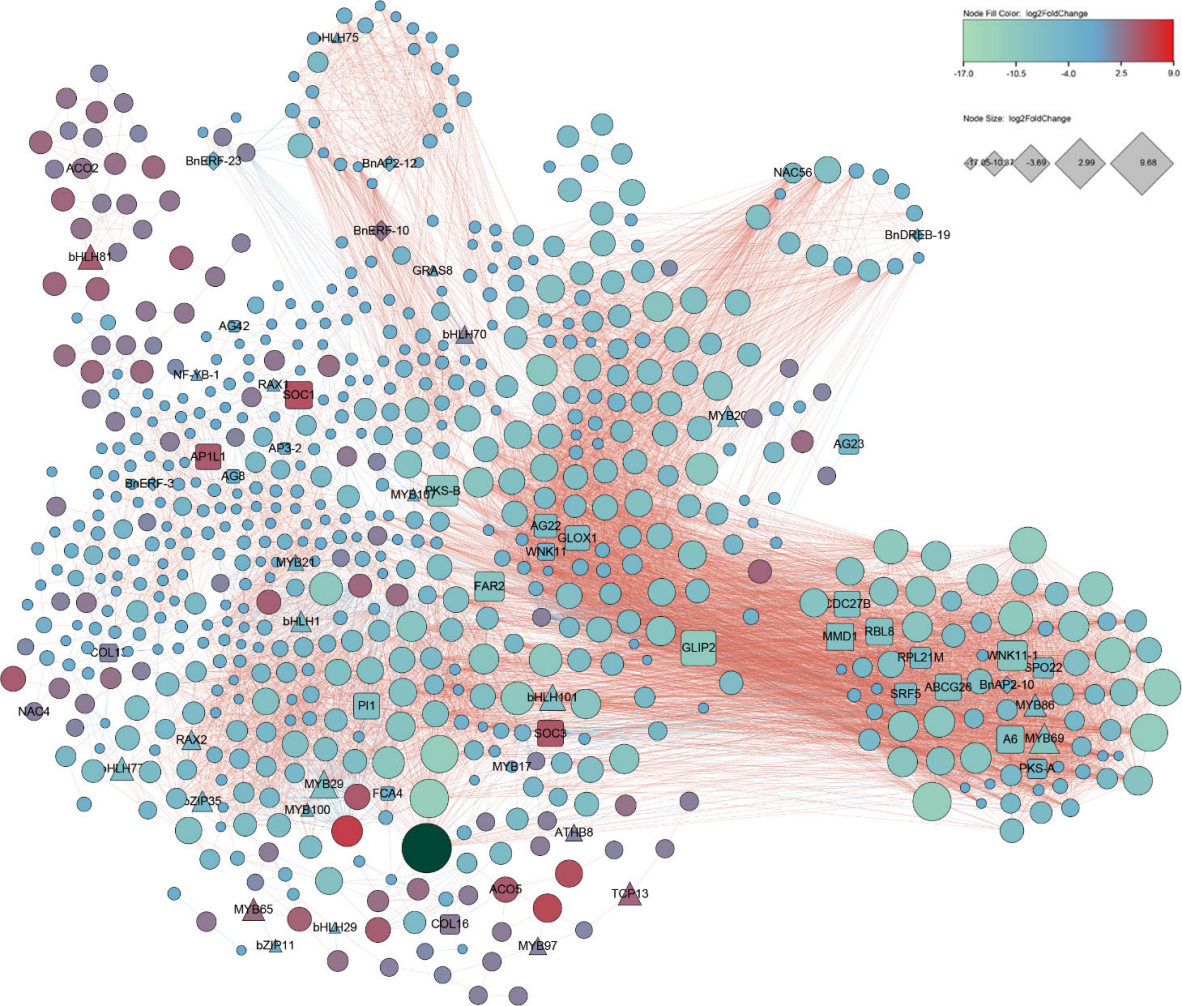
Co-expression network developed with *BnAP2/ERFs* flowering, and transcription factor genes expressed in different flower tissues. This co-expression network was constructed with DEGs with FDR absolute correlation > 0.8 and p-Value < 0.0001.

DEGs were used for network analysis to understand gene interactions during male and female flower development. We used 712 DEG candidates with FDR absolute correlation > 0.8 and p-Value < 0.0001 DEGs for Pearson correlation analysis followed for construction of a co-expression network. Of the 712 DEGs, six were BnAP2-10 (log2FoldChange=-4.3), BnAP2-12(log2FoldChange=-3.7), BnERF-10(log2FoldChange=2.6), BnERF-23(log2FoldChange=-4.9), BnERF-31(log2FoldChange=-3.0), and BnDREB-19(log2FoldChange=-2.7) from the BnAP2/ERF family (**Figure 7**; **Supplementary Table S7**). Of these six, the expression level of BnERF-10 was higher in the female flowers of GBN09 than in the male flowers of GBN08, while the expression levels of other five BnAP2/ERF genes were higher in the male flowers of GBN08 than in the female flowers of GBN09. Based on known flowering-related genes reported from *Arabidopsis* and other crops, the network included 26 flowering-related genes of ramie. The nine MADS box genes were AGL22, AGL23, AGL42, AGL8, AP1L1, AP3-2, PI1, SOC1, and SOC3. TCP13 is in the TCP family (Urano et al., 2022). Eight genes—MMD1, RBL8, GLOX1, A6, SRF5, ABCG26, PKS and FAR2—are associated with the male flower and microspore development. Two genes—RPL21M and CDC27B—are associated with female gamete development. SPO22 is a female and male flower meiosis-related gene. Five genes were related to flowering time, including two members of WNK11, FCA4, and two members of COL. Other TFs included members of the Bzip, bHLH, and MYB families. Based on the report that AeAP2/ERF061 and AeAP2/ERF067 regulate flower development of *A. eriantha* (Jiang et al., 2022), this network suggests that these BnAP2/ERF genes may participate in the regulation of flower development and flower sex differentiation of ramie.

### 3.7 Gene cloning and phylogenetic analysis of BnAP2-12

**Figure 8:**
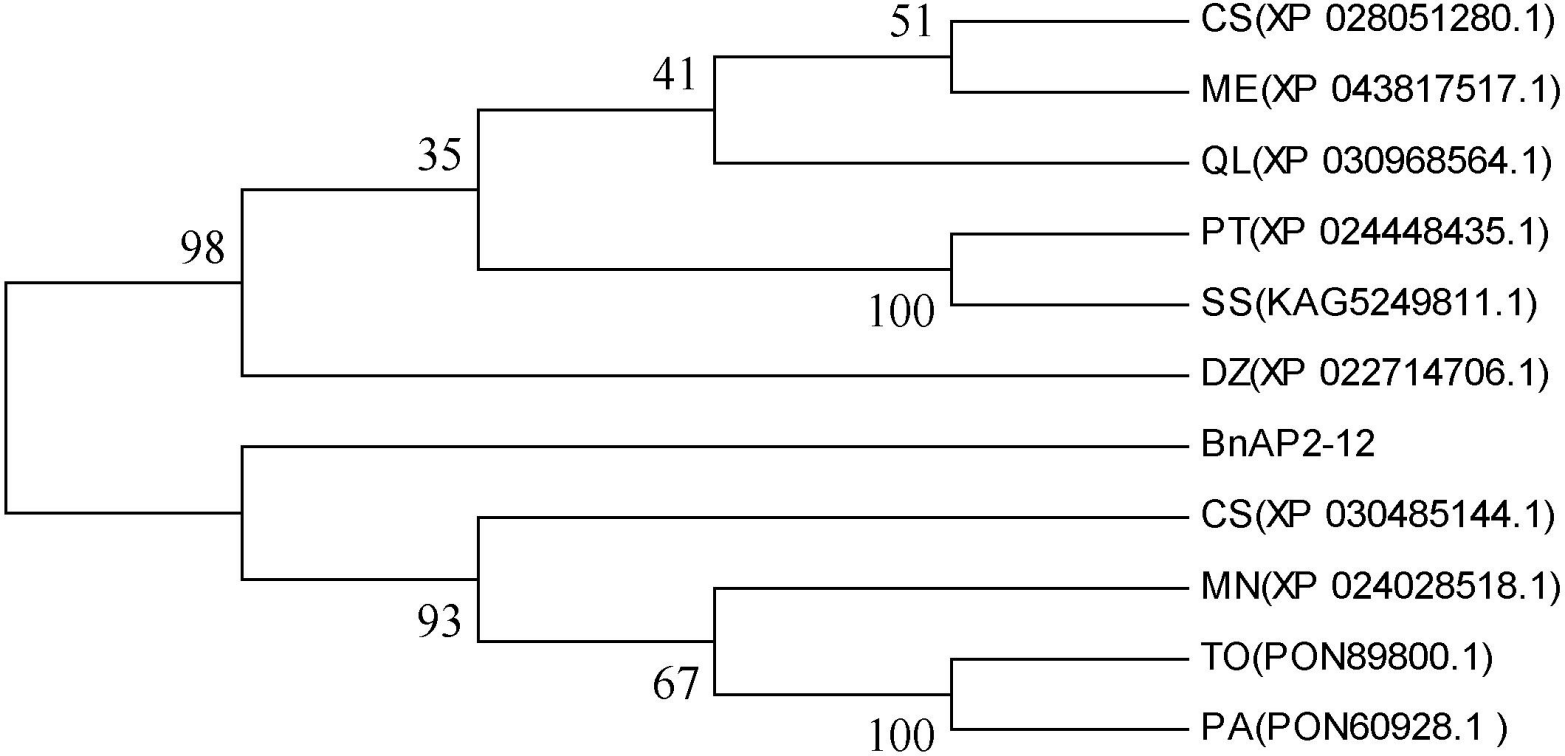
Phylogenetic tree developed with BnAP2-12 and its homologs from other 10 plant species. MN(XP_024028518.1):*Morus notabilis*; CS(XP_030485144.1): *Cannabis sativa*; PT(XP_024448435.1): *Populus trichocarpa*; TO(PON89800.1): *Trema orientale*; PA(PON60928.1): *Parasponia andersonii*; CS(XP_028051280.1): *Camellia sinensis*; ME(XP_043817517.1): *Manihot esculenta*; SS(KAG5249811.1): *Salix suchowensis*; DZ(XP_022714706.1): *Durio zibethinu*; QL(XP_030968564.1): *Quercus lobata*.

Based on the abovementioned AP2/ERF analysis, the cDNA of BnAP2-12 was cloned from our plants (**Supplementary Figure S8A**). The length of its ORF was 1113 bp, which encoded 368 amino acids. The Expasy online software predicted its molecular weight, isoelectric point, instability coefficient, lipid solubility index, and average hydrophobicity values to be 41.25, 7.15, 56.34, 59.95, and −0.840 KDa, respectively. Domain analysis identified two AP2 domains (**Supplementary Figure S8B**). Nucleotide sequence alignment indicated that the similarity between the sequences of BnAP2-12 and its homologs from *Populus trichocarpa* (XP_024448435.1), *Durio zibethinus* (XP_022714706.1), *Manihot esculenta* (XP_043817517.1), and *Cannabis sativa* (XP_030485144.1) were 77.4%, 74.31%, 73.99%, and 73.32%, respectively (**Supplementary Figure S9**). A phylogenetic tree constructed with ORF sequences showed that BnAP2-12 was clustered in its homologs from *Morus notabilis* (XP_024028518.1), *Trema orientale* (PON89800.1), *Cannabis sativa* (XP_030485144.1), and *Parasponia andersonii* (PON60928.1) (**Figure 8**).

### 3.8 Subcellular localisation of BnAP2-12

**Figure 9:**
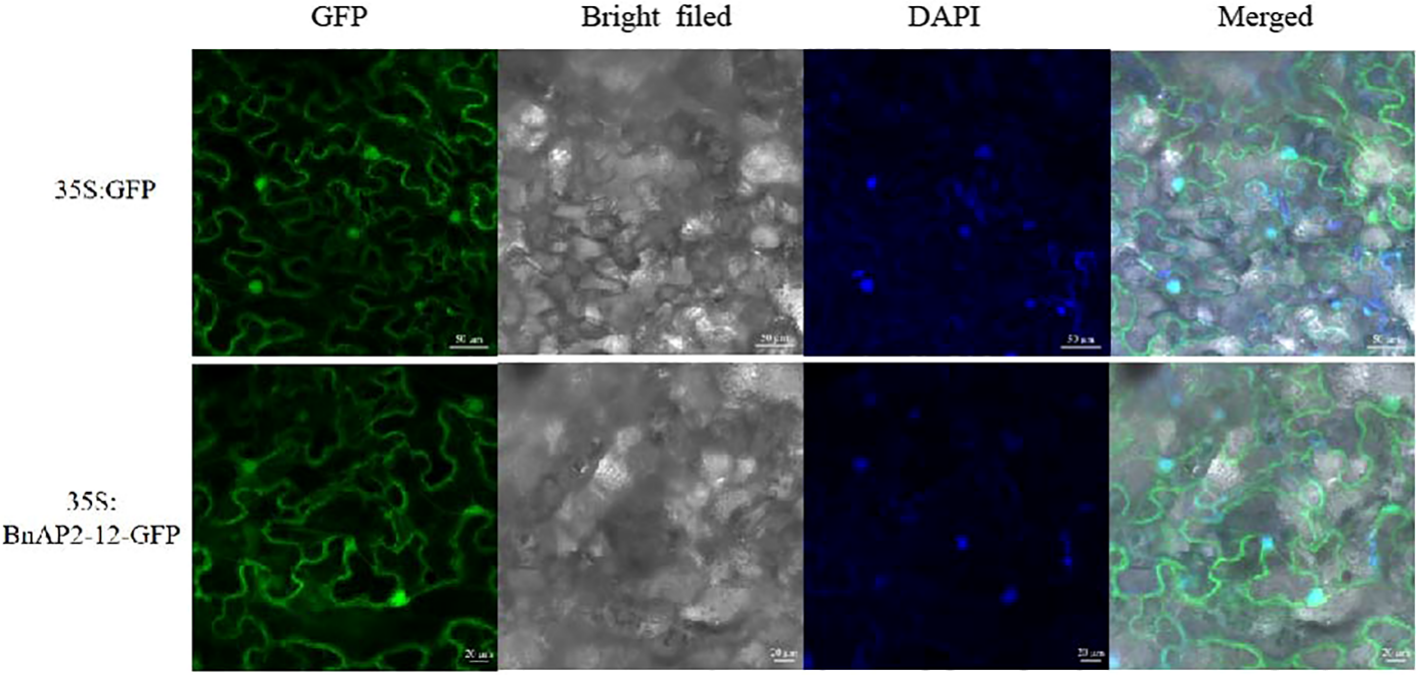
Subcellular localisation of BnAP2-12. DAPI: nuclear positioning signal.

The ORF lacking the stop codon was cloned to pCAMBIA1302, in which BnAP1-12 was fused to the N-terminus of GFP. This fusion obtained a new recombinant binary vector BnAP2-12:GFP, which was introduced into *Agrobacterium tumefaciens*. Additionally, pCAMBIA1302 was introduced into *Agrobacterium*. Two vectors were introduced into tobacco leaves through *Agrobacterium*-mediated transformation. Leaves were used for confocal microscope observation. The results showed that BnAP2-12:GFP was localised in the nucleus and on the membrane, while 35S:GFP was localised in the cytoplasm (**Figure 9**). DAPI staining as a positive control in the nuclei confirmed that BnAP2-12:GFP was localised in the nucleus.

### 3.9 Overexpression of BnAP2-12 in *Arabidopsis* delays flowering

**Figure 10:**
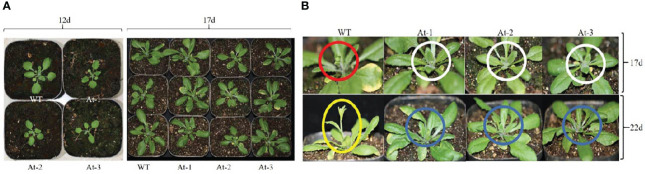
Overexpression of *BnAP2-12* delays the flowering time of Arabidopsis thaliana. Phenotypes were recorded after transplanting to soil. **(A)** images show growth phenotypes at 12 and 17 days, **(B)** Snapshots of images show flowering phenotypes between WT and transgenic plants at 17 and 24 days of plant development. Red and white circles indicate flower development from WT and transgenic plants at 17 days, respectively. WT plants were bolting, while transgenic plants did not start yet. Yellow ecliptic and blue circles show flowering between WT and transgenic plants. WT plants were in blooming, while transgenic plants just started bolting. WT, the wild-type Arabidopsis; At-1, At-2, At -3, three transgenic lines.

**Figure 11:**
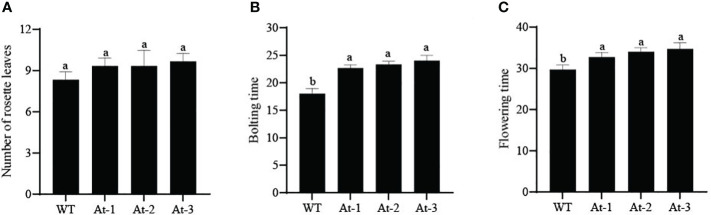
Bolting traits of WT and transgenic Arabidopsis thaliana. **(A)** Number of rosette leaves in Arabidopsis; **(B)** Bolting time in Arabidopsis; **(C)** Flowering time in Arabidopsis. WT: the wild-type Arabidopsis; At-1, At-2, At -3: three transgenic lines. Values represent means ± standard deviation (SD). Different letters in the same column indicate significant differences (P<0.05).

BnAP2-12 was introduced into *A. thaliana* for overexpression. RT-PCR analysis and antibiotic-resistance selection showed that the genetic transformation obtained multiple transgenic plants. To observe the development of flowers, T2 progeny of transgenic plants were screened to obtain homozygous T3 plants. After homozygous plants were grown in the pot soil (**Figure 10**), the growth and development were recorded daily. During the first 12 d of growth, the transgenic plants had fewer and smaller rosette leaves than wild type (WT) plants. However, with plant growth on day 17, this difference became insignificant. When plant growth entered the flowering stage, transgenic plants exhibited different phenotypes. When WT *Arabidopsis* plants started to bolt inflorescence, all other transgenic plants did not. On day 22, WT *Arabidopsis* plants bloomed, while transgenic plants had just started to bolt inflorescences. When transgenic plants started to bloom, WT plants started to develop siliques. Statistical analysis of flowering time revealed that the BnAP2-12 overexpressing strain flowered on around the 34^th^ day, approximately 5 d later than the wild type, reaching the level of highly significant difference. Statistical analysis of the time of shoot extraction and number of rosette leaves revealed no significant difference between the number of rosette leaves of the BnAP2-12 overexpression strain and the wild type at the time of first flowering, but the wild type extracted shoots 4 d earlier than the BnAP2-12 overexpression strain (**Figure 11**). These results showed that the overexpression of BnAP2-12 delayed the flowering time of *Arabidopsis*. These data indicate that BnAP2-12 controls the flowering time.

## 4 Discussion

Genomics and transcriptomics empower studies of ramie development for fibre and leaf production. Ramie is an important natural fibre and forage crop in China. Improvement of the vegetative growth is crucial for biomass and livestock. Given that early flowering can reduce plant biomass, breeding ramie cultivars with late- or non-flowering has been an important agricultural goal for high fibre and leaf yields. Unlike model plants, ramie plants are a perennial species featuring unisex flowers and monoecious, androecious, or gynoecious flowers. It has been challenging to use general mutagenesis methods to create genetic materials. Despite this problem, we collected numerous ecotypes and used hybridization to create multiple cultivars for fibre or high protein production. GBN08 and GBN09 are two cultivars bred for high production of fibres and leaf biomass. Both have unique flowering phenotypes, which provide appropriate materials to study male and female flower differentiation. In this study, we took advantage of a ramie genomic sequence to use bioinformatic tools to mine genes associated with flowering and flower development. Additionally, based on multiple reports, AP2/ERF transcription factors play an important role in flowering regulation and development in model plants, such as rice and *Arabidopsis* (Jofuku et al., 1994; Nakano et al., 2006; Tanaka et al., 2014). Although these AP2/ERF TF members were reported in studies on waterlogging and the ramet development of ramie (Qiu et al., 2022), no AP2/ERFs were found to be involved in the regulation of flowering and development. Our data provide novel insights into the roles of AP2/ERF TF in flower development. In addition, we found that BnAP2-12 was overexpressed in *A. thaliana*; however, it is not possible to extrapolate these results to the sexual development of ramie. Specifically, the dynamics of BnAP2-12 remain to be studied in ramie (i.e., through transgenic plant systems).

Based on the genome of the ramie cultivar ZSZ No. 1 (Wang et al., 2021), we identified 84 AP2/ERF genes, less than that found in an earlier study on a different ramie variety (Zhongzhu No1). This discrepancy likely resulted from two different cultivars. To understand whether AP2/ERFs are involved in the flower development of gynoecious germplasm GBN09 and monoecious germplasm GBN08, we assembled transcriptomes of male and female flowers. These data allowed us to annotate AP2/ERF and other TFs associated sexual differentiation. Several studies have reported on plants related to flowering and floral organ development. The AGL, PI, and AP3 (Agamous-like MADS-box protein) genes play multiple roles in plant development (Guo et al., 2016; Malabarba et al., 2017; Montiel et al., 2020). In cucumbers, the expression of AP1L1 (CsMADS09) decreases during the development of male flowers but remains almost unchanged during the development of female flowers. The ectopic expression of AP1L1 leads to early flowering and abnormal leaf development in transgenic *Arabidopsis* (Zhou et al., 2019). In maize, SOC1 overexpression can lead to early flowering and increased yield (Han et al., 2021). In this study, transcriptome differential analysis showed that the genes WNK11 (Bni04T005400) and FCA4 (Bni11T016163), which are related to flowering time, are expressed at low levels in GBN09 ramie, whereas COL genes (Bni04T006470, BniUnT019294) are highly expressed. Similarly, our results showed that 50% of the MADS genes related to flower organ composition were highly expressed in GBN08, whereas the other 50% of the MADS genes, including AP1L1 (Bni10T014882) and SOC1 (Bni08T012449 and Bni08T012453), were expressed at low levels in GBN08 (**Supplementary Table S6**). Whether the high expression of this gene causes GBN-08 and GBN09 to bloom earlier will require further investigation. Additionally, DEGs used for network analysis was analysed for clustering on the MCODE plug-in with a cutoff degree value of 3 and Clusters with a score > 5 were taken for expression analysis (**Supplementary Table S8** and **Supplementary Figure S10**). Twelve clusters comprising 369 DEGs were found to be the most significant. Most DEGs in 12 clusters were lowly expressed in female flowers in GBN09, except for 21 genes (**Supplementary Table S10**). In the context of sexual differences, these genes were most expressed in males than in females, suggestive of their synergistic promotion of the development of male flowers and inhibitory effect on the development of female flowers. Further studies will be required to clarify these trends.

Gene expression and functional patterns are closely related (Lee et al., 2017). The AP2/ERF family of genes plays a crucial role in the development and reproduction of flowering plants. Studies show that these genes are highly expressed in flowers and are involved in the regulation of ethylene-mediated flowering induction, floral organ development, and plant growth (Zhang L. et al., 2017; Guyomarc’h et al., 2021). Recently, a study on pineapple revealed functional differences in ethylene-mediated flowering induction and floral organ development; the researchers identified 97 AP2/ERF family members in the pineapple genome and divided them into five subfamilies. The results showed that the ERF and RAV subfamilies may have important roles in the ethylene reaction of pineapple and that the ERF and DREB subfamily genes have special functions in flower organ development (Zhang et al., 2021). In addition to pineapple, other plant species, such as *Medicago truncatula* and rice, have also been studied to understand the role of the AP2/ERF gene family in plant development. MtERFs are highly expressed in the flowers, buds, roots, nodules, blades, and seedpods of *M. truncatula* (Shu et al., 2015). In rice, expression levels in the roots, ears, and flowers are higher than those in the leaves and stems (Rashid et al., 2012). These results indicate that the AP2/ERF gene family is related to plant development and flowering reproduction and are highly expressed in flowers. In ramie, the expression of different AP2/ERF gene families in the flowers of different sexes is specific. For example, BnAP2-12 and BnAP2-10 were significantly expressed in flowers and their expression levels in male flowers were considerably higher than those in female flowers. However, BnERF-14 and BnERF-40 showed opposite expression patterns. These findings suggest that the ERF and AP2 subfamily genes of ramie have special functions in the development of different flower organs. These results will have significant implications for crop production and plant breeding.

Transcriptome sequencing offers the possibility of studying the relationship between plant traits and the function of a particular class of genes. The differential gene analysis and selection of a gene with significantly different expression dynamics for functional analyses can potentially yield clear trait-gene associations. In this study, BnAP2-12 was selected for cloning and shown to be overexpressed in *Arabidopsis thaliana*. In addition, this gene was found to regulate flowering in *Arabidopsis thaliana*. As the flowering process is important for enhancing the genetics of plants (Hassankhah et al., 2020), we propose that BnAP2-12 may be a regulator of flowering. The ectopic expression of AfAP2-2 in *Arabidopsis* has been shown to delay flowering, specifically under short day (SD) conditions (Lei et al., 2019). Multiple studies have shown that AP2/ERF transcription factors are key regulators of the timing of flowering in plants. For example, when the AP2/ERF transcription factor TOE4b is overexpressed in soybeans, not only is flowering delayed but the physical appearance of the plant is also altered, resulting in a higher grain yield per plant (Li et al., 2023). In chrysanthemum, the interaction between CmERF110 and FLOWERING LOCUS KH DOMAIN (CmFLK) promotes flowering (Huang et al., 2022). In this study, we created *A. thaliana* lines that overexpressed this gene and observed a delay in flowering in the T3 lines. However, further research is required to fully understand the specific mechanisms underlying flowering. Although this study revealed that significantly differentially expressed gene (BnAP2-12) can regulate flowering time in *Arabidopsis*, uncertainty remains regarding the revealed gene-trait associations. Overall, more comprehensive and fine-scale genomic analyses will be necessary to.

## 5 Conclusions

AP2/ERF genes in the ZSZ1 ramie genome were systematically analysed using bioinformatic tools and algorithms. For the 84 identified BnAP2/ERF genes, four subfamilies were defined and categorised. Conserved motifs, structural chromosome distributions, and cis-acting regulatory element analyses were performed. Fragment replication events were identified as the main forces underlying BnAP2/ERF development in synteny and collinearity studies. The transcriptome data showed 1,803 and 1,832 upregulated and downregulated genes, respectively, between the male and female flowers. GO enrichment analysis revealed that pollen formation and female gamete formation-related genes were significantly enriched. Examination of KEGG pathways revealed that these DEGs were predominantly involved in metabolic pathways and the formation of secondary metabolites. A total of 22 BnAP2/ERFs existed that were significantly upregulated, and only eight were significantly downregulated in female flowers. Co-expression network analysis showed that six AP2/ERF genes were involved in the co-expression network, including BnAP2-12. The BnAP2-12 coding sequence was cloned and subcellular localisation analysis showed that it was localised in the nucleus and cell membrane. Furthermore, overexpression of BnAP2-12 was found to delay *A. thaliana* flowering. The results of this study suggest that the BnAP2/ERF genes are crucial for ramie flowering.

## Data availability statement

The datasets presented in this study can be found in online repositories. The names of the repository/repositories and accession number(s) can be found in the article/**Supplementary Material**.

## Author contributions

XZ: Conceptualization, Data curation, Formal analysis, Investigation, Visualization, Writing – original draft, Writing – review & editing. WP: Conceptualization, Data curation, Investigation, Resources, Visualization, Writing – original draft, Writing – review & editing. HC: Data curation, Supervision, Validation, Writing – review & editing. HX: Conceptualization, Funding acquisition, Investigation, Resources, Validation, Writing – original draft.
