# Supplementary material for: BnAP2-12 overexpression delays ramie flowering: evidence from AP2/ERF gene expression
Source: Front Plant Sci. 2024 Mar 25;15:1367837. doi: 10.3389/fpls.2024.1367837 (PMC10999622; doi:10.3389/fpls.2024.1367837)
Supplement: Supplementary file 1 [file DataSheet_1.docx]

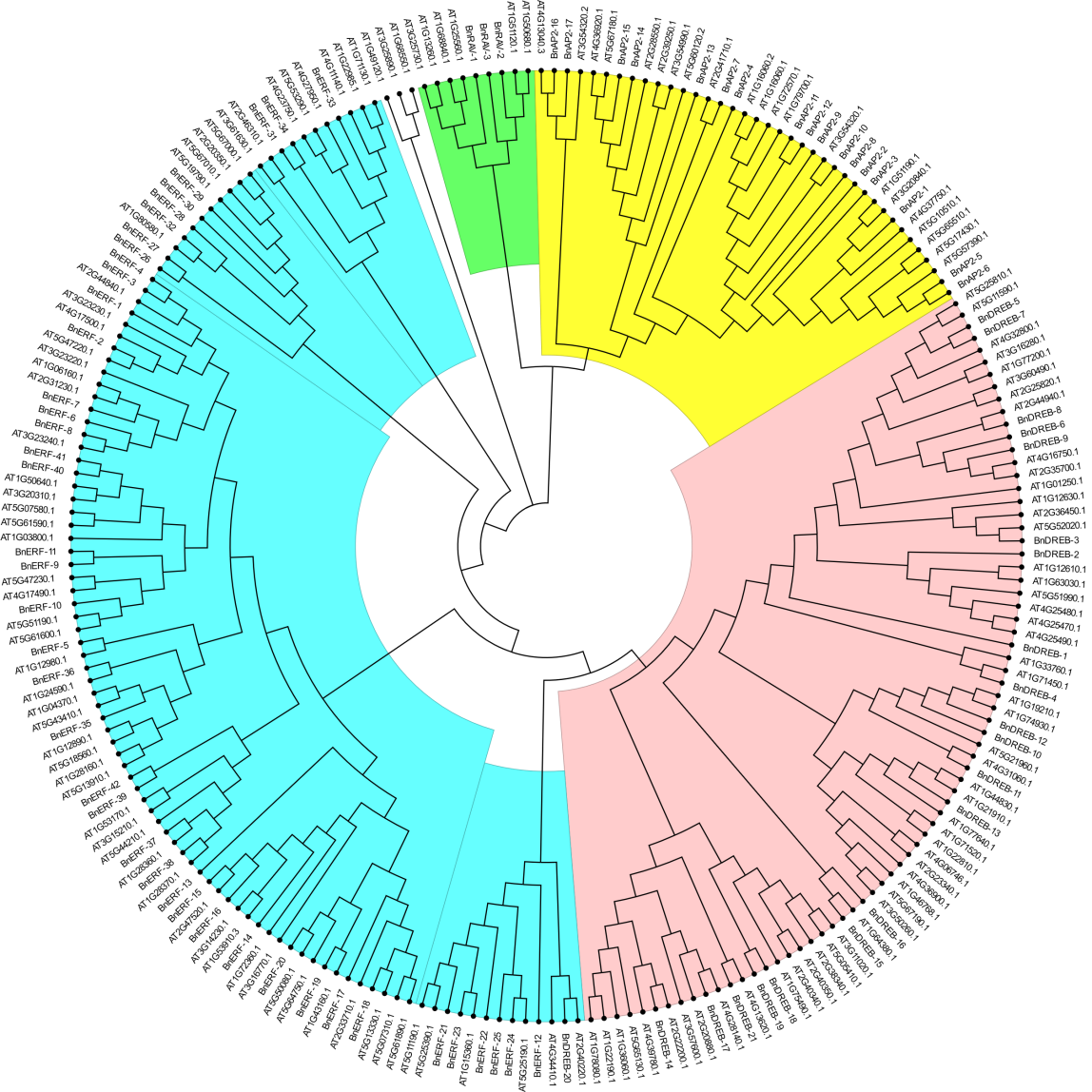


Figure S1. Phylogenetic analysis of 84 ramie AP2/ERF proteins. A phylogenetic tree by the maximum likelihood (ML tree) method was constructed using muscle software for the multiple sequence alignment of 84 AP2/ERF proteins sequences of ramie and 147 AP2 / ERF proteins sequences in Arabidopsis (yellow: AP2-type; blue: ERF-type; pink: DREB-type; green: RAV-type;). The bootstrap consensus tree inferred from 1000 replicates is taken to represent the evolutionary history of the taxa analyzed. Branches corresponding to partitions reproduced in less than 50% bootstrap replicates are collapsed.
