# Supplementary material for: BnAP2-12 overexpression delays ramie flowering: evidence from AP2/ERF gene expression
Source: Front Plant Sci. 2024 Mar 25;15:1367837. doi: 10.3389/fpls.2024.1367837 (PMC10999622; doi:10.3389/fpls.2024.1367837)
Supplement: Supplementary file 2 [file DataSheet_2.docx]

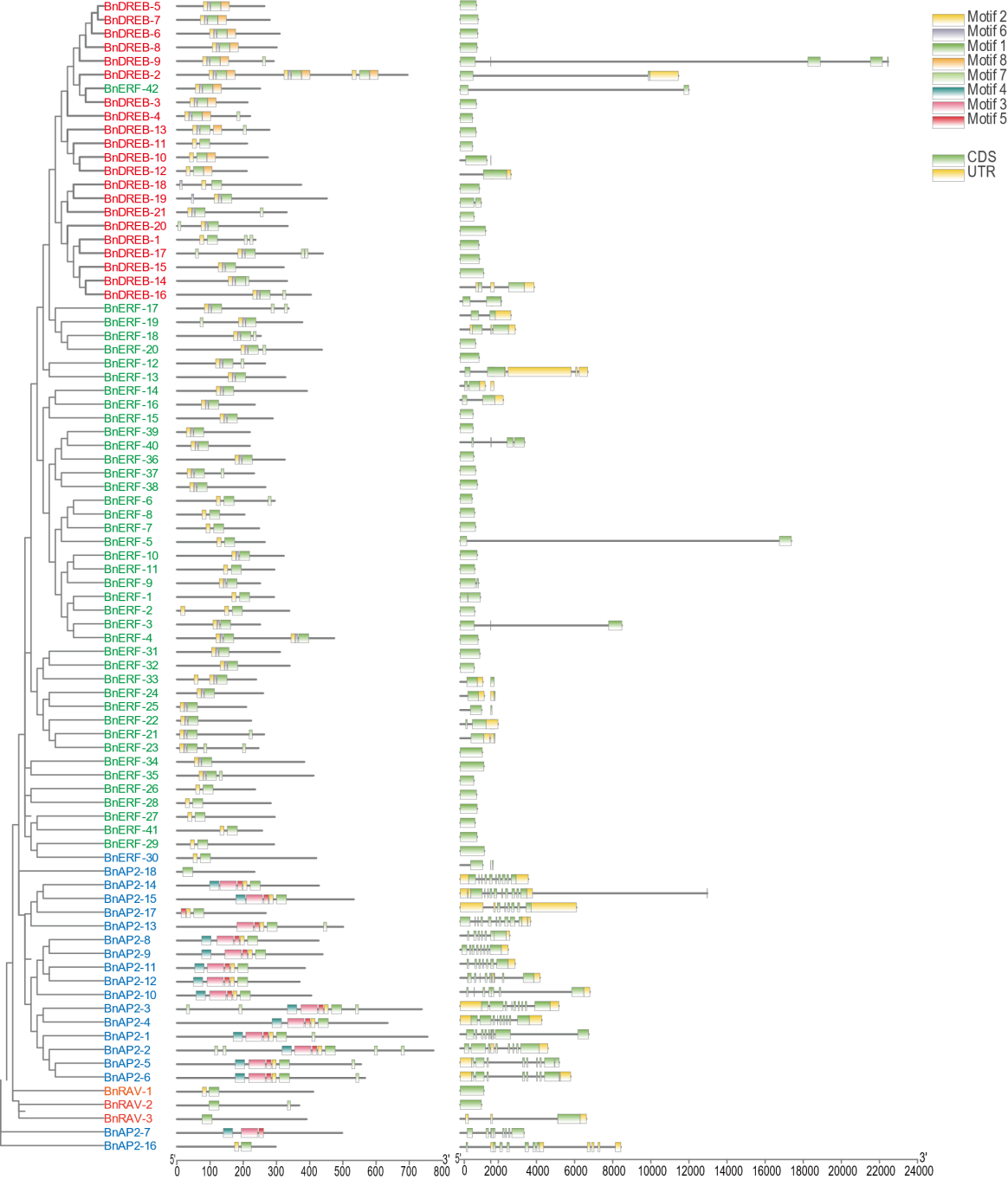


Figure S2. Distribution of conserved motifs and gene structure in BnAP2/ERF proteins. Distribution of conserved motifs according to MEME motif analysis. Differently conserved motifs are indicated with colored boxes. Distribution of gene structure according to GSDs analysis. The sequences of each motif are presented in Supplementary table S3.
