# Supplementary material for: BnAP2-12 overexpression delays ramie flowering: evidence from AP2/ERF gene expression
Source: Front Plant Sci. 2024 Mar 25;15:1367837. doi: 10.3389/fpls.2024.1367837 (PMC10999622; doi:10.3389/fpls.2024.1367837)
Supplement: Supplementary file 3 [file DataSheet_3.docx]

**
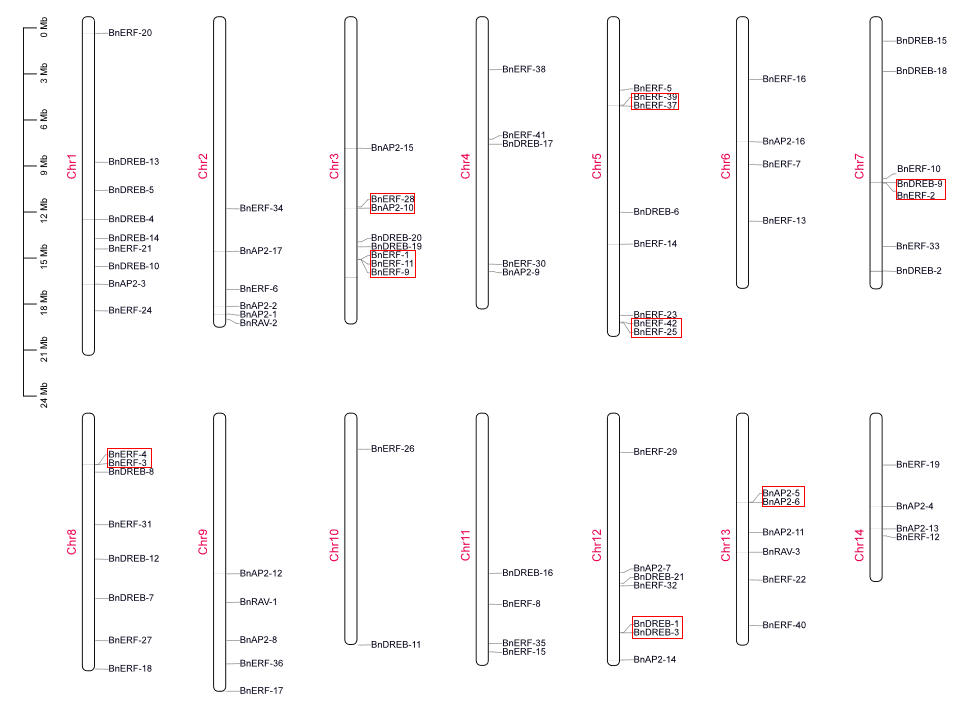
**

Figure S3. Chromosomal locations of BnAP2/ERFs. The gene name is presented to the right of each bar, while the chromosome name is to the left. To the left of chr1 is a 3-Mb bar. The clusters are shown with a red frame.
