# Supplementary material for: BnAP2-12 overexpression delays ramie flowering: evidence from AP2/ERF gene expression
Source: Front Plant Sci. 2024 Mar 25;15:1367837. doi: 10.3389/fpls.2024.1367837 (PMC10999622; doi:10.3389/fpls.2024.1367837)
Supplement: Supplementary file 4 [file DataSheet_4.docx]

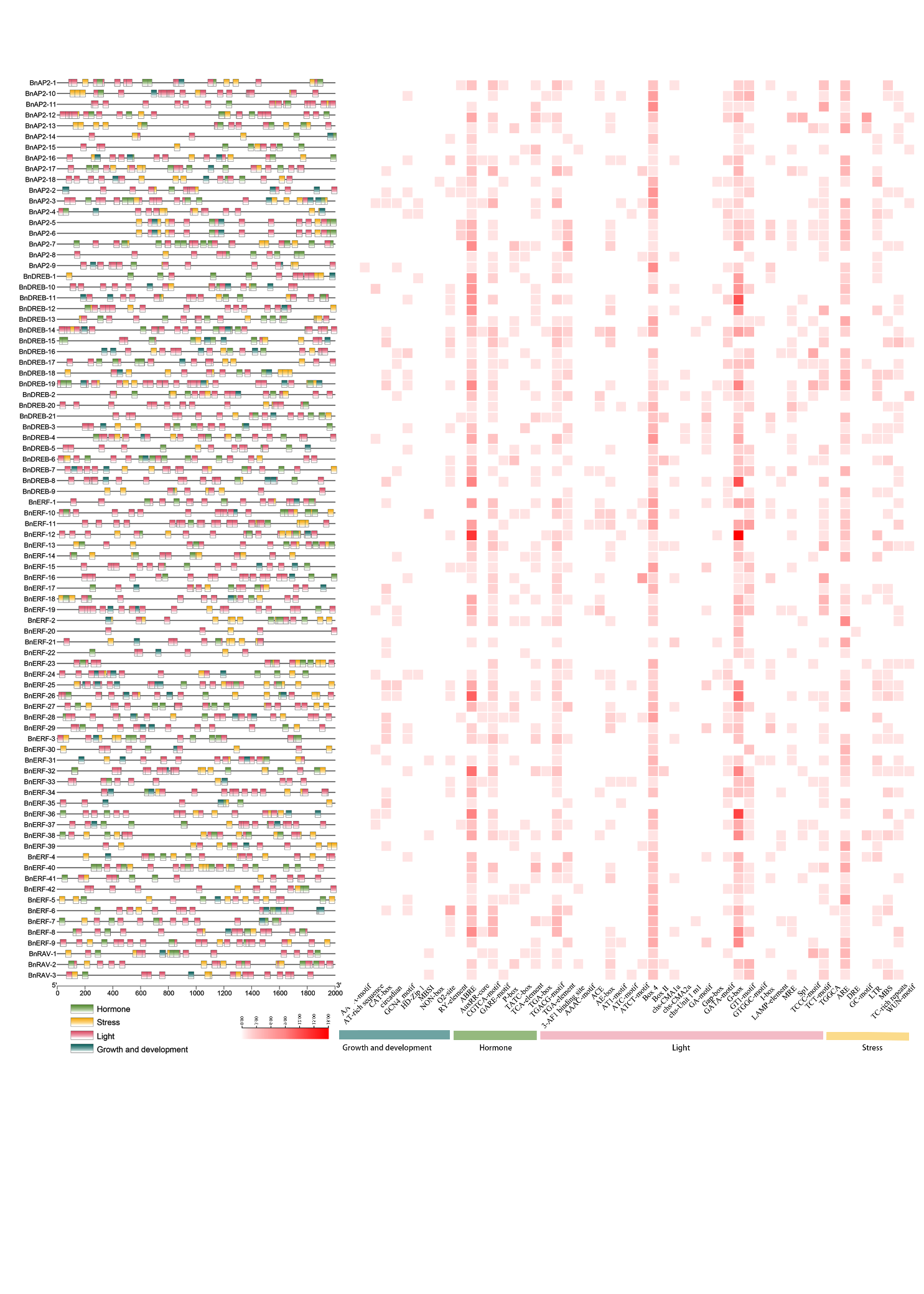


Figure S4. Analysis of cis-acting elements in the promoter region of BnAP2/ERFs. The left panel shows the distribution of cis-acting elements in the promoter region. The heat maps of cis-acting elements for the light-responsive, hormone-responsive, stress-responsive, growth and development-responsive, and the color concentration of the squares indicates the number of cis-acting elements.
