# Supplementary material for: BnAP2-12 overexpression delays ramie flowering: evidence from AP2/ERF gene expression
Source: Front Plant Sci. 2024 Mar 25;15:1367837. doi: 10.3389/fpls.2024.1367837 (PMC10999622; doi:10.3389/fpls.2024.1367837)
Supplement: Supplementary file 5 [file DataSheet_5.docx]

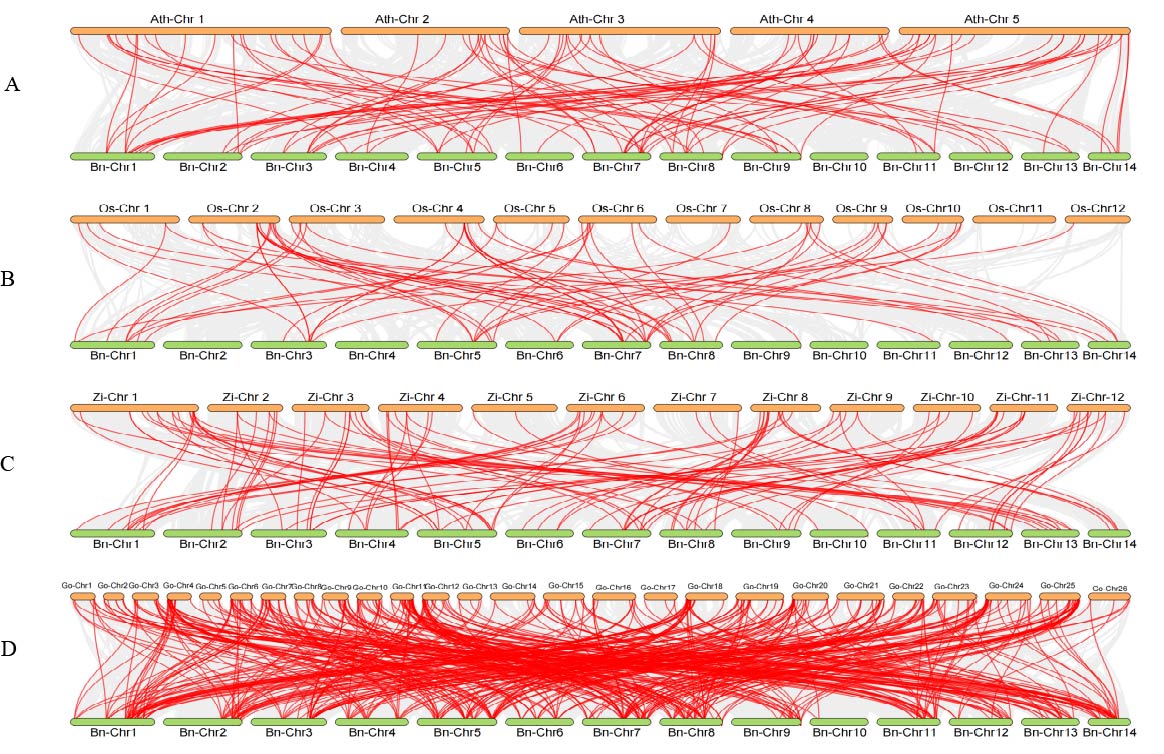


**Figure S5. Collinearity and synteny relationship of *BnAP2/ERF* genes between ramie and Arabidopsis, rice, jujube, and cotton.** Collinear gene pairs are developed between ramie and Arabidopsis (A), rice (B), jujube (C), and cotton (D). The gray line in the background shows the collinear area and the red line area highlights the collinear gene pair.
