# Supplementary figures and images for: BnAP2-12 overexpression delays ramie flowering: evidence from AP2/ERF gene expression
Source: Front Plant Sci. 2024 Mar 25;15:1367837. doi: 10.3389/fpls.2024.1367837 (PMC10999622; doi:10.3389/fpls.2024.1367837)

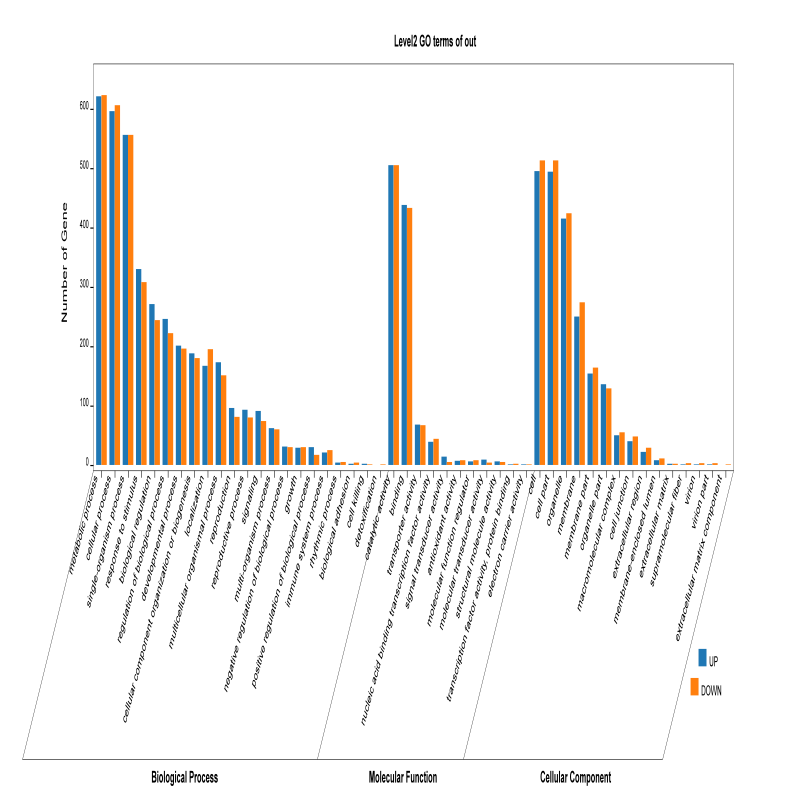


Figure S6. GO enrichment functional annotation and classification of DEGs

Supplement: Supplementary file 6 [file DataSheet_6.docx]

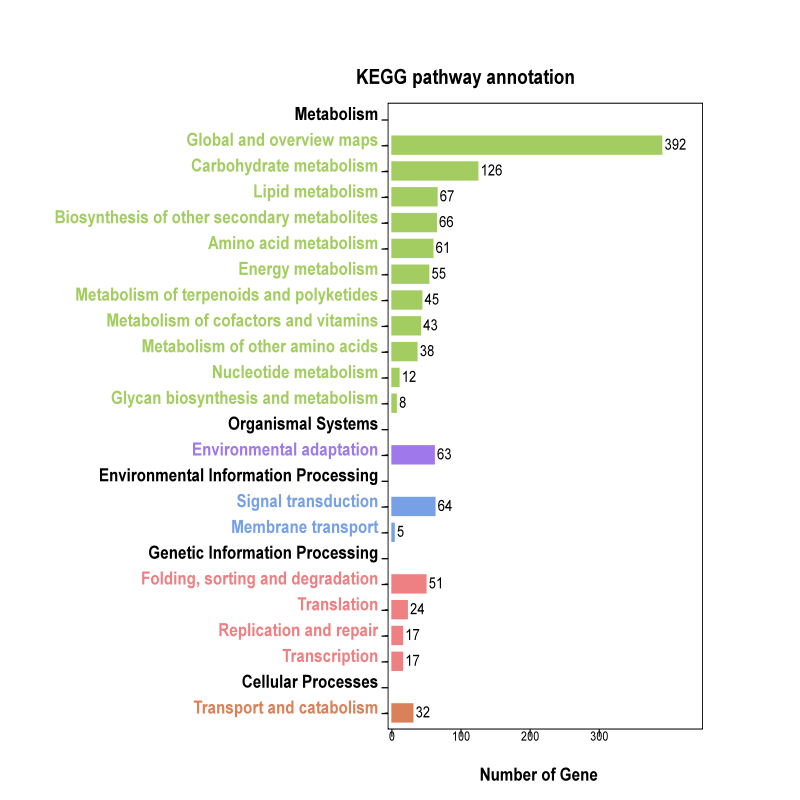


Figure S7. KEGG pathway annotation and classification of DEGs

Supplement: Supplementary file 7 [file DataSheet_7.docx]

**
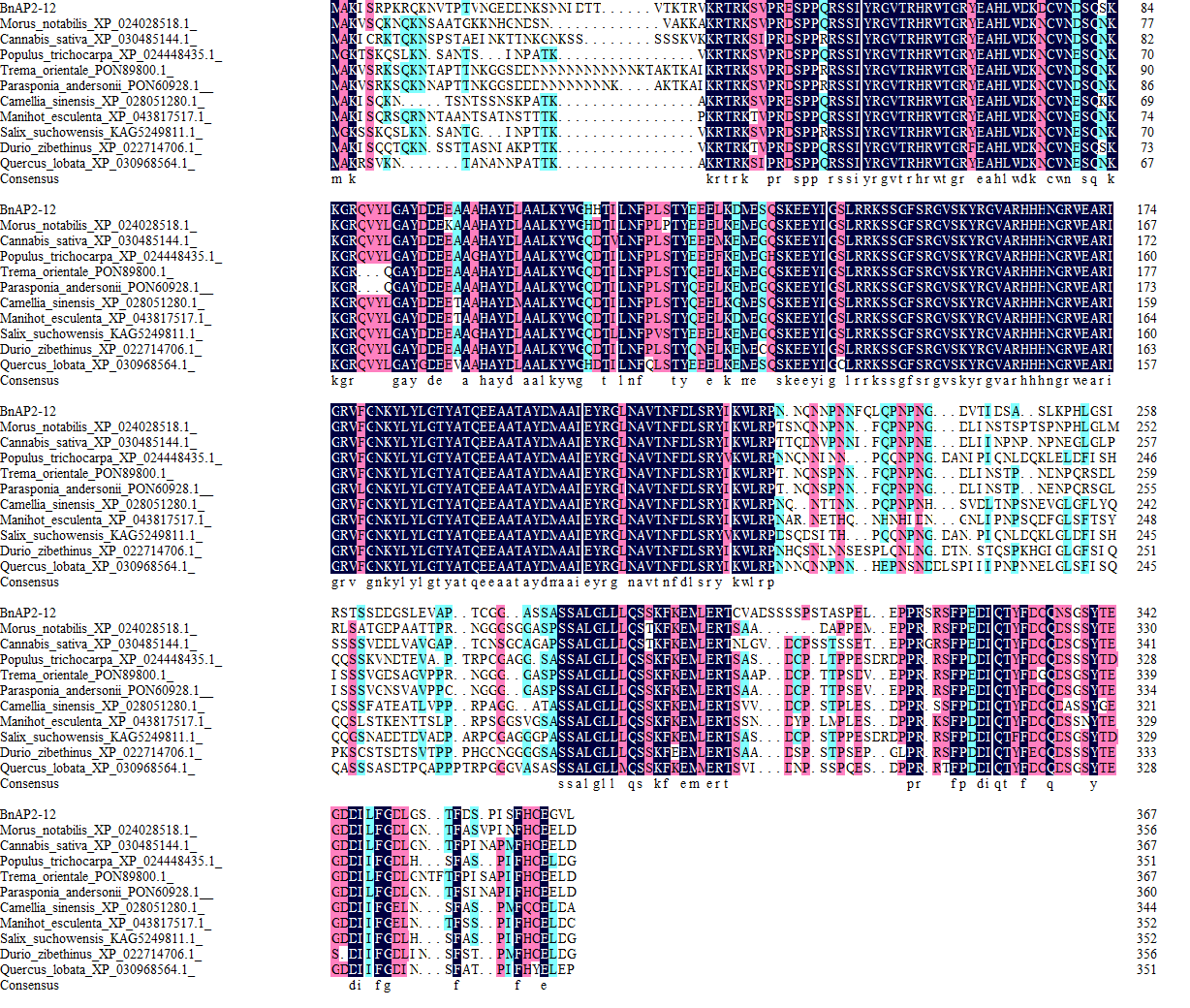
**

Figure S9 Sequence alignment of ramie BnAP2-12 with other species.

Supplement: Supplementary file 9 [file DataSheet_9.docx]

**
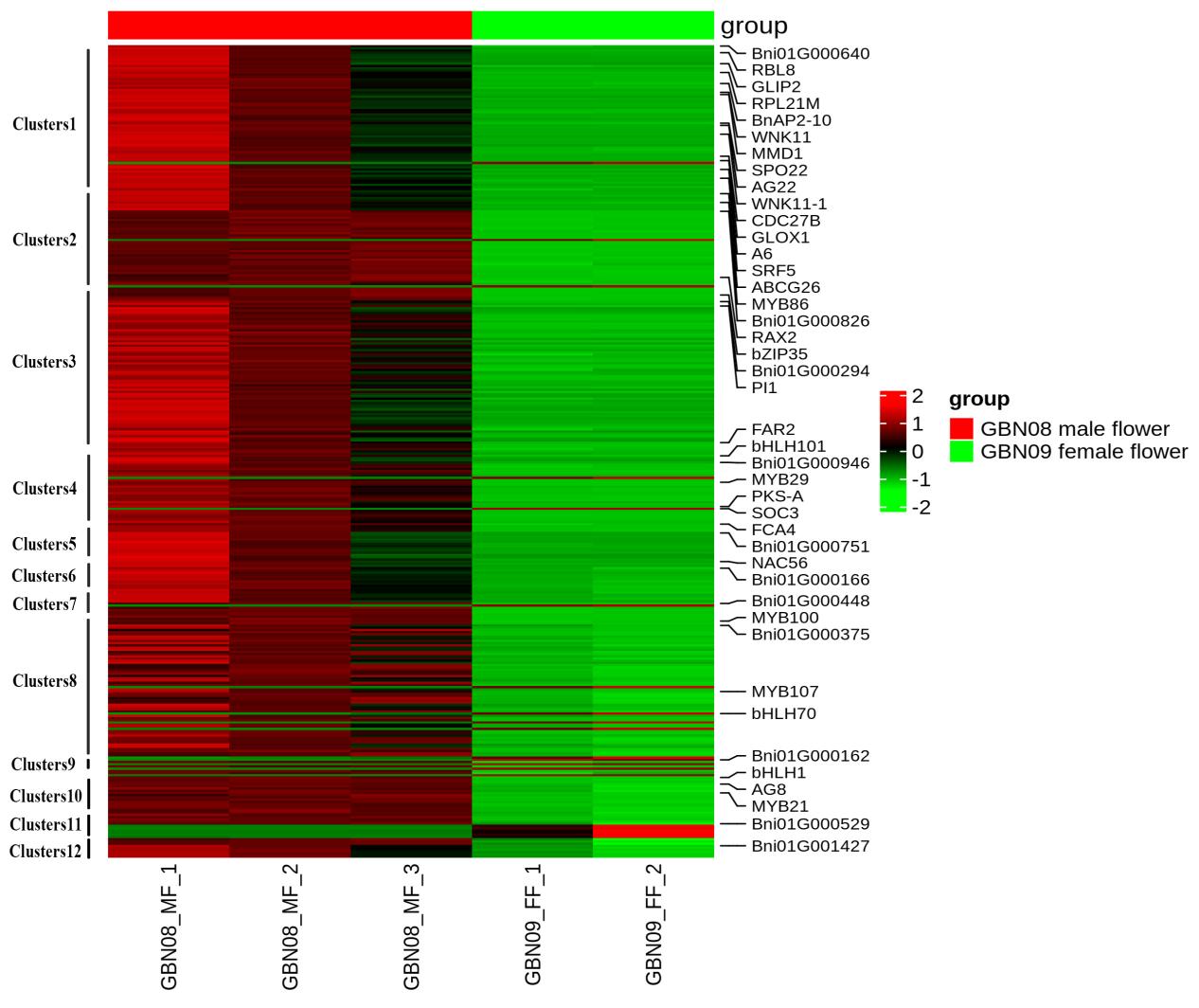
**

Figure S10 Expression profiles of clustered genes in different sexual flowers.

Supplement: Supplementary file 10 [file DataSheet_10.docx]
