# Supplementary material for: BnAP2-12 overexpression delays ramie flowering: evidence from AP2/ERF gene expression
Source: Front Plant Sci. 2024 Mar 25;15:1367837. doi: 10.3389/fpls.2024.1367837 (PMC10999622; doi:10.3389/fpls.2024.1367837)
Supplement: Supplementary file 8 [file DataSheet_8.docx]

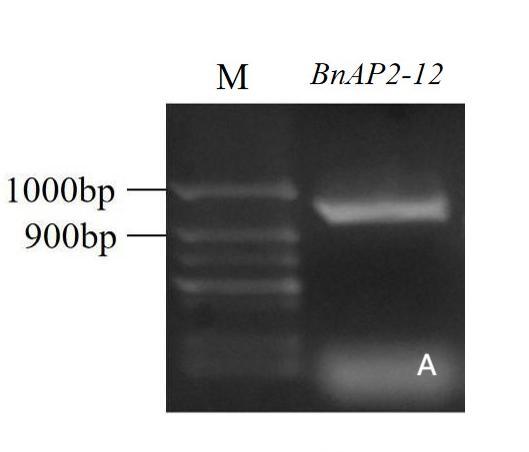

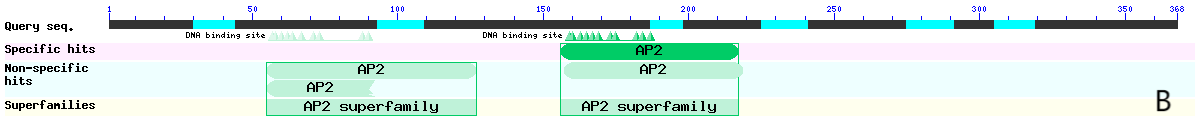


Figure S8 Electropherogram of BnAP2-12 CDS and conservative domain analysis.

(A)Horizontal gel electropherogram of PCR production.(B)Conservative domain analysis
